# Towards the Development of Standardized Bioassays for Corals: Acute Toxicity of the UV Filter Benzophenone-3 to Scleractinian Coral Larvae

**DOI:** 10.3390/toxics10050244

**Published:** 2022-05-10

**Authors:** Ingo B. Miller, Mareen Moeller, Matthias Y. Kellermann, Samuel Nietzer, Valentina Di Mauro, Elham Kamyab, Sascha Pawlowski, Mechtild Petersen-Thiery, Peter J. Schupp

**Affiliations:** 1 Environmental Biochemistry, Institute for Chemistry and Biology of the Marine Environment (ICBM), Carl von Ossietzky University Oldenburg, Schleusenstr. 1, 26382 Wilhelmshaven, Germany; mareen.moeller@uni-oldenburg.de (M.M.); matthias.kellermann@uni-oldenburg.de (M.Y.K.); samuel.nietzer@uni-oldenburg.de (S.N.); valentina.di.mauro@uni-oldenburg.de (V.D.M.); elham.kamyab@uni-oldenburg.de (E.K.); 2 Department of Product Safety, Regulatory Ecotoxicology, BASF SE, Carl-Bosch-Str. 38, 67056 Ludwigshafen am Rhein, Germany; sascha.pawlowski@basf.com; 3 Product Stewardship & EHS Data Management, BASF Personal Care and Nutrition GmbH, 40789 Monheim am Rhein, Germany; mechtild.petersen-thiery@basf.com; 4 Helmholtz Institute for Functional Marine Biodiversity, University of Oldenburg (HIFMB), Ammerländer Heerstr. 231, 26129 Oldenburg, Germany

**Keywords:** coral reefs, ecotoxicology, organic ultraviolet filters, marine pollution, early life history, short-term bioassay, standardization, oxybenzone, settlement, sunscreen

## Abstract

Coral reefs have been declining globally at a historically unprecedented rate. Ultraviolet (UV) filters used in sunscreens may contribute to this decline at local scales, which has already led to bans on various organic UV filters in some regions. However, the underlying studies for these bans demonstrated significant flaws in the experimental design due to a lack of validated and standardized testing methods for corals. This study aimed to investigate options for the development of a standard acute toxicity test for the larval stage of scleractinian corals. Planula larvae of two brooding (*Leptastrea purpurea* and *Tubastraea faulkneri*) and two spawning (*Acropora digitifera* and *A. millepora*) species were exposed to the organic UV filter benzophenone-3 (BP3) for 48 h under static conditions. We observed interspecific variations in toxicity, with *A. digitifera* being the most sensitive (LC_50_ = 0.75 µg L^−1^) and *T. faulkneri* the least sensitive (LC_50_ = 2951.24 µg L^−1^) species. Inhibition of settlement was found to be a useful endpoint leading to an EC_50_ of 1.84 µg L^−1^ in *L. purpurea* larvae. Although the analytical challenges of measuring lipophilic substances in small volume test setups remain, the here applied test design and selected endpoints are suitable for further validation and subsequent standardization.

## 1. Introduction

Tropical coral reefs provide a wide array of essential ecosystem services such as coastal protection, fisheries and tourism to human societies around the globe [1,2]. They cover less than 0.1% of the seafloor [3] but harbor at least one quarter of all known marine species with many reef species yet to be discovered [4]. Owing to their highly efficient nutrition retention and cycling capacity, hermatypic corals thrive in oligotrophic warm water environments [5,6,7], transforming these otherwise unproductive marine areas into biodiversity hotspots [8].

Regardless of their high value for mankind, various human activities are progressively pushing coral reefs towards the tipping point of functional collapse [9,10,11,12,13,14]. On a global scale, the increase in sea surface temperatures (SST) driven by climate change is the most alarming threat to tropical reefs [15,16]. In fact, marine heat waves have been found to be the primary cause of widespread coral bleaching events and coral loss in recent decades [17]. Recurring and prolonged bleaching events have already occurred between 2014–2017 and critically affected global coral reefs [9,18,19,20]. Under the current emissions scenario, severe, annually recurring bleaching is projected to occur by mid-century [21]. Besides climate change-related effects, a wide range of additional human-induced stress factors such as overfishing and destructive fishing [22,23,24], pollution and coastal development contribute to coral reef decline on a local scale [2]. Given the multitude of stressors, it is suggested that local action should be taken to minimize such direct impacts on coral reefs [11,14,25,26,27,28,29,30].

In recent years, sunscreen products and their active ingredients, ultraviolet (UV) filters, have gained scientific and public attention, as they may also impact coral health on a local scale due to their direct release into coastal marine areas through recreational activities (e.g., swimming, snorkeling) and sewage systems [31]. While UV filters in sunscreen products are important aids to protect humans from harmful UV radiation [31,32,33,34], several studies demonstrated negative health effects and enhanced mortality to corals after exposure to various widely used UV filters [35,36,37,38,39,40,41,42,43,44,45,46,47,48]. For example, Downs et al. [36] reported mortality in coral larvae of *Stylophora pistillata* a LC_50_ (i.e., concentration that kills 50% of the population) at nominal 139 µg L^−1^ after exposure to the organic UV filter benzophenone-3 (BP3 or oxybenzone). Based on these studies, policymakers in various locations (i.e., Hawaii, US Virgin Islands, Palau, Aruba and Bonaire) have already taken regulatory actions and banned the use of some organic UV filters including BP3, ethylhexyl methoxycinnamate (EHMC or octinoxate), octocrylene (OCR) and 4-methylbenzylidene camphor (4MBC) in sunscreens [49,50,51]. Those bans, however, are controversial as the underlying studies revealed several shortcomings (e.g., lack of analytical verification of exposure concentrations, inadequate controls, lack of environmental relevance) that question the reliability of some of the test results and the conclusions drawn within these studies [49,50,51,52]. Several recent review articles have compiled a comprehensive overview about the current status of coral toxicity research on UV filters and their shortcomings [48,49,51]. Therefore, these results may be considered as preliminary and require further scientific evaluation, preferably conducted using standardized testing methods on corals including various relevant life stages [49,53]. To this end, standardized toxicity protocols that include different life stages of corals have yet to be developed.

Most studies carried out so far investigated the substance-related toxicity of UV filters on adult corals, whereas only a few considered the larval stage [36,40] and none considered early recruits [49]. However, early life stages (i.e., planula larvae, early post-settlement recruits) are suggested to be generally more sensitive to environmental stressors and chemical pollutants, compared to adults [54,55,56,57]. Therefore, stress occurring early in life can lead to severe structural impacts on populations and communities [58,59]. In fact, the inhibition of larval settlement and/or metamorphosis, which are crucial developmental steps and ultimately determine survival, have been documented for the herbicide diuron [60], the antifouling agent tributyltin [61,62] and other organic pollutants, including a range of hydrocarbons [63,64,65,66,67].

Recently, several reviews discussed relevant aspects to be considered in coral toxicity assays including different life stages [49,50,53]. Accordingly, and to address the outlined issues, we investigated acute toxic responses of scleractinian planula larvae (i.e., free swimming larvae from stony coral) exposed to the organic UV filterBP3 under standardized laboratory conditions. For this, (I) planula larvae of two broadcast spawning (i.e., release of eggs and sperm and fertilization in water column [68]) corals (i.e., *Acropora millepora* and *Acropora digitifera*) and two brooding (i.e., internal fertilization and release of competent planulae [68]) corals (i.e., *Leptastrea purpurea* and *Tubastraea faulkneri*) were exposed to BP3 for 48 h in acute bioassays under static exposure conditions. Here, we performed a larval survival assay on all four coral species and an additional larval settlement assay on the two brooding species. The suitability of different toxicity endpoints (i.e., mortality and/or inhibition of settlement) were assessed. Furthermore, analytically verified concentrations were used to (II) estimate biological endpoints (i.e., 50% lethal and effect concentrations, LC_50_/EC_50_, respectively).

In line with previous findings for other organic compounds [60,61,62,63,64,65,66,67], we hypothesize that UV filters might inhibit the settlement process in coral larvae, and that settlement could be an useful endpoint for toxicity testing. This study will contribute towards the development of standardized coral toxicity tests for scientifically sound decision-making processes by authorities. Furthermore, those tests will also help producers of UV filters and formulators of sunscreens in selecting more ecofriendly candidates for both existing and future substances and products in order to contribute to a better safeguard of the already fragile coral reef environment.

## 2. Materials and Methods

### 2.1. Chemicals

Benzophenone-3 (BP3; 98%, CAS No. 131-57-7) was obtained from Sigma-Aldrich (Taufkirchen, Germany). Bis(tri-n-butyltin) oxide (TBTO; 97%, CAS No. 56-35-9, abcr GmbH, Karlsruhe, Germany) was used as positive control. Ethanol (EtOH; ≥99.8%, Carl Roth GmbH & Co. KG, Karlsruhe, Germany) was used as solvent to spike BP3 in water samples for matrix-matched calibrations. Tetrachloroethylene (TCE; HPLC grade, ≥99.9%, Sigma-Aldrich, Taufkirchen, Germany) was used as extraction solvent and formic acid (FA; Biosolve BV, Valkenwaard, The Netherlands) for adjusting the pH of water samples. For the analytical system, acetonitrile (ACN; ULC/MS grade, ≥99.99%; Biosolve BV, Valkenwaard, The Netherlands), and MilliQ water (ultrapure water purification system arium 611DI, Sartorius AG, Göttingen, Germany), both containing 0.01% FA, were used. A synthetic salt mix (Pro-Reef salt, Tropic Marin, Prof. Dr. Biener GmbH, Wartenberg, Germany) was used for the preparation of artificial seawater. Further details on physicochemical properties [69,70,71] of the active ingredients (BP3, TBTO), and chemicals used in additional bioassays can be found in Tables S1 and S22.

### 2.2. Coral Species and Acquisition of Larvae

Toxicity experiments with *L. purpurea* and *A. digitifera* were conducted after the July 2019 spawning event in the Western Pacific at the facilities of the University of Guam Marine Laboratory (UOGML), Guam, USA. The collection of colonies was carried out under the ‘Special License for the Collection of Coral’ issued to the UOGML by the Guam Department of Agriculture. Gravid colonies (n = 8, ca. 25 cm in diameter) of the hermatypic broadcast spawning coral *A. digitifera* were collected in 3–5 m depth from the reef crest at Pago Bay (13°25′35″ N, 144°47′47″ E) prior to the spawning event and held in 4000 L tanks sustained by an open natural seawater flow-through system. During the spawning in July, gamete bundles were collected from the water surface, and subsequently, sperm and eggs were mechanically separated. Cross-fertilization was achieved by mixing gametes from several *A. digitifera* colonies. After fertilization, embryos were transferred into larval culture tanks from where planula larvae were collected for the larval assays after developing into competent planulae.

A broodstock of mature *L. purpurea* colonies (n = 100, ca. 5–10 cm in diameter) was collected for larvae acquisition in 1–2 m depth from the reef flat at Luminao Reef (13°27′56″ N, 144°38′48″ E) according to Nietzer et al. [72]. Briefly, colonies were held in 400 L flow-through tanks (as described above) and fed every other night (with a blended mix of smelt, mussel, squid and shrimp), while water supply was switched off for one hour during feeding. Temperature and light intensity in the tanks were monitored using data loggers (HOBO Pendant UA-002-64, Onset Computer Corporation, Bourne, MA, USA). Colonies were placed on plastic grates for simple handling. Before sunset, colonies were transferred into collection tanks (approx. 25 L) where larvae were released overnight. Water circulation and gas exchange was maintained by three 6 mm air tubings at an air flow rate of at least 60 mL min^−^^1^ (approximately 3 bubbles s^−1^) each. The next morning, water from the collection tanks containing the released larvae was filtered through a 30 µm mesh and the collected larvae were carefully transferred into a glass bowl. Owing to the presence of a green fluorescent protein in *L. purpurea* planulae [72,73], we collected competent larvae under fluorescence blue light (wavelength 440–460 nm; BlueStar™, Nightsea, Lexington, MA, USA) and a yellow barrier filter (longpass) using a Pasteur pipette. Upon collection, larvae were transferred into glass beakers containing 0.22 µm filter sterilized seawater (FSW) and held in an incubator at natural sea water condition of 29 °C.

Experiments with *A. millepora* and *T. faulkneri* were carried out at the indoor aquarium facilities of the Institute for Chemistry and Biology of the Marine Environment (ICBM) at the University of Oldenburg, Wilhelmshaven, Germany. Water supply was sustained through a closed ex situ recirculating system with artificial seawater made from a synthetic salt mix (Pro-Reef salt, Tropic Marin, Prof. Dr. Biener GmbH, Wartenberg, Germany) that was dissolved in reverse osmosis water at 39 g L^−^^1^. Gravid colonies of the hermatypic broadcast spawning coral *A. millepora* (imported from the northern Great Barrier Reef, Australia (CITES permit No: 20NL284183/11) in November 2020) were kept in two 600 L tanks in an ex situ spawning facility (designed and constructed by Coral Spawning Lab, Ltd., Morden, London, UK) for a two-week acclimatization phase prior to the anticipated spawning event in early December 2020. Abiotic parameters relevant for spawning synchronicity (i.e., lunar cycle, day length, water temperature) were simulated in accordance with natural conditions following a recently established protocol [74]. After cross fertilization from 8 conspecific colonies (ca. 8–10 cm in diameter), embryos were transferred to larval culture tanks (as described above for *A. digitifera*). Brooding *T. faulkneri* colonies (n = 6, ca. 10 cm in diameter) were imported from Indonesia (CITES permit No: 20NL288025/11) and kept in 250 L tanks within the 6500 L recirculating system. Released planulae were collected using a Pasteur pipette and transferred into glass beakers containing FSW at 26 °C. See Table S2 for detailed overview of conditions for the husbandry of adult coral species.

### 2.3. Preparation of Test Media

Water was collected from the same culture tanks (natural seawater and artificial seawater for bioassays conducted in Guam and in Germany, respectively) as the respective parent corals and filter-sterilized through a 0.22 µm nitrocellulose membrane filter before being used for bioassays (dilutions and controls) and for the preparation of stock solutions (target compound dissolved in seawater). For simplification, we subsequently refer to filtered artificial and filtered natural seawater as filtered seawater (FSW). The latter was used as a negative media control (NC) in the bioassays.

Individual stock solutions of nominal 12 mg L^−^^1^ and 140 mg L^−^^1^ in FSW were prepared in Schott glass bottles by direct addition [75], reflecting twice the known solubilities of BP3 and TBTO in pure water (cf. Tables S1 and S4). They were then stirred for 24 h at room temperature to prepare saturated stock solutions, followed by a resting period of 1 h to assure phase separation between dissolved and undissolved fractions of the test material. To avoid particulate test material in the final stock solution, about 80% of the solution was then pipetted carefully from the center of the water body and transferred to a clean glass bottle. Stock solutions were freshly prepared before each experimental run and stored in the absence of light at 4 °C until use. Nominal concentrations of the saturated stock solutions were adjusted to the known solubility limits in distilled water (BP3: 6 mg L^−^^1^, TBTO: 70 mg L^−^^1^). Note that the latter adjustment is an approximation, as actual water solubility limits in seawater may differ.

### 2.4. Acute Larvae Toxicity Assays

The acute toxicity of BP3 on coral larvae from *A. digitifera, A. millepora, L. purpurea* and *T. faulkneri* was investigated in a 48 h static toxicity test using 12-well cell culture plates (polystyrene; TPP Techno Plastic Products AG, Trasadingen, Switzerland), similar to a previously introduced protocol [76,77]. A stepwise 2-fold serial dilution of the stock solution (nominal 6 mg L^−^^1^) was prepared directly in 12-well cell culture plates. The controls (negative: FSW, positive: nominal 0.7 mg L^−^^1^ TBTO) and each treatment group were run in triplicates. Five planulae were carefully transferred with 500 µL FSW into each well using a 1000 µL pipette. The well plates were stored in an incubator equipped with LED light panels at ambient temperature (29 °C for *L. purpurea* and *A. digitifera*, and 26 °C for *T. faulkneri* and *A. millepora*) in a 12 h day/12 h night light cycle for a total period of 48 h. For all four species, a survival experiment was conducted to assess the mortality rates of planula larvae after direct exposure to the compound.

To assess potential inhibitions of the settlement process from exposure to the compound, settlement experiments were additionally conducted for the two brooders *L. purpurea* and *T. faulkneri*. For this, crustose coralline algae (CCA) of the species *Hydrolithon reinboldii*, collected from Luminao Reef (13°27′56″ N, 144°38′48″ E) in Guam, USA, were used as a settlement inducer [72,73]. A CCA chip (ca. 0.5 cm^3^) was placed into each well before five larvae per well were introduced. The negative control in the settlement assays consisted of FSW and a CCA chip. After 48 h exposure, the endpoint ‘settlement’ was assessed in addition to ‘mortality’ using a dissection microscope (Olympus SZ40, 6.7–40× magnification; Olympus Corporation, Tokyo, Japan). For experiments with *L. purpurea*, fluorescent blue light in combination with a yellow barrier filter (longpass) was used to spot swimming and/or settled larvae.

**Figure 1:**
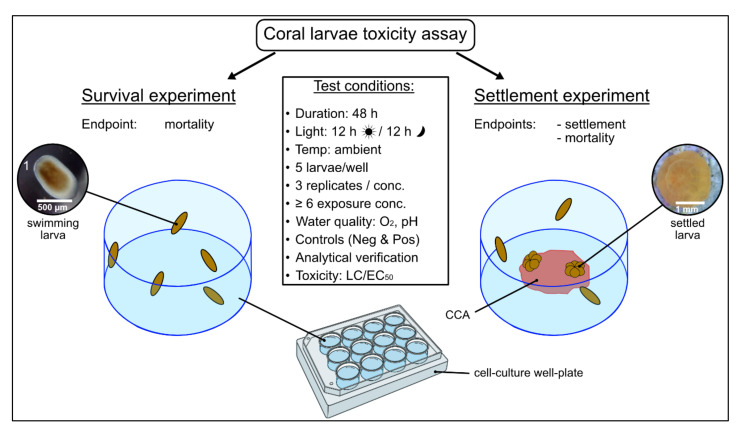
Schematic overview of the acute toxicity assay performed using coral larvae conducted in 12-well cell culture plates. The endpoint ‘mortality’ was assessed in the survival experiment, while ‘settlement’ (metamorphosis + attachment) was assessed additionally in the settlement assay. Planula larvae were exposed to the target compound for 48 h to assess acute toxic responses to the compound. A crustose coralline algae (CCA) chip was used to induce settlement. See Table S3 for more details on the exact condition during the experiments. ^1^ Photo courtesy of L. Fiegel.

Larvae were considered dead when there was no visible movement, and/or cellular disintegration was present. Larvae were defined as settled when they had attached to substrate (CCA chip or on the bottom/walls of the well) and started developing into a coral recruit (i.e., metamorphosis and attachment). At this stage, pronounced flattening of the oral-aboral axis and the development of septal mesenteries radiating from the central mouth region were obvious [76]. A schematic overview of the acute bioassays is illustrated in Figure 1.

Preliminary assessments were conducted to find a suitable range of exposure concentrations. Accordingly, six nominal concentrations ranging from 83.3 to 1.3 µg L^−^^1^ were chosen for the survival experiments with *L. purpurea* and *A. digitifera*. Due to limited larval supply over a longer time period, survival experiments with *T. faulkneri* and *A. millepora* were conducted without prior range finding tests in a 13 step dilution series ranging from 5.3 mg L^−^^1^ to 1.3 µg L^−^^1^. Based on the results from the survival experiments, the settlement assays with *L. purpurea* were conducted in nominal concentrations ranging from 166.7 to 1.3 µg L^−^^1^ (n = 7), while *T. faulkneri* larvae were exposed to six BP3 concentrations ranging from 5.3 mg L^−^^1^ to 166.7 µg L^−^^1^.

For a test to be considered valid, we defined the following criteria: The negative controls should not exceed a mortality rate of 20% and settlement should be ≥70%. Dissolved oxygen should be maintained at ≥80% air saturation (corresponds to 5.4 mg DO L^−^^1^ seawater with 35‰ salinity at 25 °C) throughout the test.

### 2.5. Water Quality

Dissolved oxygen (DO) and pH levels were monitored at the start and the end of each assay. For assays conducted in Guam, pH was measured using a commercially available digital pH meter, and DO was measured using a titration test kit (Oxygen Test Kit, Salifert Europe B.V., Duiven, The Netherlands) for assays with *A. digitifera*, while a digital DO probe (HI98193, Hanna Instruments, Woonsocket, RI, USA; accuracy ± 1.5%) was used for *L. purpurea* experiments. For assays conducted in Germany (*A. millepora* and *T. faulkneri*), a multi-parameter portable meter (WTW MultiLine 3630 IDS, Xylem Analytics, Weilheim, Germany) coupled with a dissolved oxygen probe (FDO 925; accuracy ± 1.5%) and a pH probe (SenTix 940; accuracy ± 0.004) was used. Measurements were taken in the stock solutions for reference conditions at the start of each experiment, and in the controls and one representative test concentration after 48 h.

### 2.6. Chemical Analysis for Verification of Exposure Concentrations

#### 2.6.1. Sampling and Sample Preparation

For analytical verification of the actual exposure concentrations, 10 mL of sample water (pooled in equal proportions from the three replicates) from at least three test concentrations (highest, medium, and lowest) was transferred into 20 mL glass vials (Wheaton Science Products, Millville, NJ, USA) at the end of each experiment (after 48 h) and stored at −20 °C prior to analysis. Likewise, BP3 stock solutions were additionally sampled at the beginning of the assays. Water samples from experiments conducted in Guam were transported to the ICBM at the University of Oldenburg, Germany for analysis.

The extraction procedure was based on a protocol implemented by Zhang and Lee [78] and modified for the use of seawater samples and analysis with UPLC-MS. For this, the defrosted water samples were acidified to pH 4 using FA, and 100 µL of TCE was added to each sample in order to achieve a 100-fold pre-concentration. Samples were then shaken for 1 min at 2500 rpm on a vortex mixing device (RS-VA 10, Phoenix Instruments GmbH, Garbsen, Germany) to ensure complete mixing and transfer of the target analyte to the extremely hydrophobic organic solvent TCE (cf. Supplementary Information (SI) S1, Figure S1 and Table S5 for detailed information on extraction efficiency in relation to vortex duration). To achieve full phase separation, the mixtures were centrifugated for 10 min at 3000 rpm (Sigma 3-16KL; Sigma Laborzentrifugen GmbH, Osterode am Harz, Germany). An aliquot of the extract (~70 µL) was then transferred from the droplet at the bottom of the vial to a 250 µL glass insert (neochrome, neoLab Migge GmbH, Heidelberg, Germany) for analysis.

For quantification of BP3 concentrations in experimental samples, calibration standards containing BP3 were used. For external (or solvent) calibration, a standard stock solution of BP3 dissolved in TCE was prepared at a concentration of 1 mg mL^−1^, from which a six-step dilution series (i.e., 10, 5, 1, 10^−^^1^, 10^−^^2^ and 10^−^^3^ mg L^−1^) was prepared in 2 mL amber glass vials (neochrome ND9, neoLab Migge GmbH, Heidelberg, Germany) and stored at −20 °C until use. A matrix-matched calibration was performed to consider the influence of the seawater matrix as well as the extraction procedure. For this, a standard stock solution of 10 g L^−1^ of BP3 dissolved in EtOH was prepared. A nine-step dilution series (i.e., 10, 5, 1, 10^−^^1^, 10^−^^2^, 10^−^^3^, 10^−^^4^, 10^−^^5^ and 10^−^^6^ g L^−1^) was prepared in 2 mL amber glass vials and subsequently, 10 µL of each dilution was added to 10 mL of acidified (pH = 4) FSW in 20 mL glass vials (Wheaton Science Products, Millville, NJ, USA) to reach the respective target concentrations (i.e., 10, 5, 1, 10^−^^1^, 10^−^^2^, 10^−^^3^, 10^−^^4^, 10^−^^5^ and 10^−^^6^ mg L^−1^). The extraction procedure for the matrix-matched calibration was performed in accordance with the experiment samples as described above.

#### 2.6.2. Analytical System

The chromatographic analyses were performed using an ACQUITY Ultra Performance Liquid Chromatography (UPLC) system (Waters Co., Milford, MA, USA), equipped with a Waters ACQUITY quaternary solvent manager, a sample manager (autosampler) set to 10 °C, a HT column heater set to 40 °C and a photo diode array detector operated between 190 and 800 nm. In general, 1 µL of each analyte was injected onto a Waters BEH C18 column (1.7 µm, 2.1 × 50 mm) with a constant flow rate of 0.6 mL min^−^^1^ for separation. A linear gradient was applied, consisting of MilliQ water (H_2_O, eluent A) and acetonitrile (ACN, eluent B), both acidified with 0.1% formic acid (*v/v*). The initial condition was 100% A for 0.5 min followed by a linear increase to 100% B within 3 min and held for 2 min. Subsequently, conditions were returned to the initial conditions (100% eluent A) and held for 1.5 min to equilibrate the column for the next run.

The UPLC system was coupled to a SYNAPT G2-Si high-definition Quadrupole Time-of-flight (QToF) mass spectrometer (MS) for mass detection (Waters Co., Milford, MA, USA) fitted with a Lock Spray dual electrospray ion source operated in positive (POS; ESI^+^) ionization mode. The QToF-MS was calibrated in sensitivity mode over a mass to charge (*m/z*) ranging from 50 to 1200 Da using a 0.5 mmol L^−1^ sodium formate solution. Data were centroided and mass was corrected during acquisition using a 1 ng µL^−1^ leucine enkephalin (C_28_H_37_N_5_O_7_) solution at a flow rate of 8 µL min^−1^ as external lock mass for each run, generating a positive reference ion ([M+H]^+^ 556.277 Da). Thus, a mass accuracy of less than 1 ppm could be achieved. Capillary voltage was 0.8 kV in POS-mode, sampling cone voltage was 80 V. Source and desolvation gas temperature was set to 150 °C and 50 °C, respectively. Cone gas (N_2_) flow was 50 L h^−1^ and desolvation gas (N_2_) flow was 1200 L h^−1^. Mass spectral data were collected using the full spectrum mode (MS1) to obtain information on the intact molecule.

#### 2.6.3. Identification and Quantification of BP3

Spectral data analysis and peak integration was performed using MassLynx Mass Spectrometry software (V4.2, Waters Co., Milford, MA, USA). The protonated molecule ion (*m/z* 229.086, [M+H]^+^) and a major in source fragment ion (*m/z* 151.040) of BP3 were used for peak quantification. Therefore, the calibration curves (external and matrix-matched) were constructed based on the peak area (PA) of the summarized signal of the extracted ions *m/z* 229.086 + *m/z* 151.040 versus analyte concentration (Figure S2). Since the measurements were performed on separate days, two calibrations were utilized. The resulting curves exhibited satisfactory linearities ranging from 1 µg L^−1^ to 5 mg L^−1^ (*R^2^* = 0.9987 and 0.9993) and 10 ng L^−1^ to 5 mg L^−1^ (*R^2^* = 0.9994 and 0.9995) for the external and matrix-matched calibrations, respectively (Figure S3). The precision, expressed as relative standard deviation (RSD) calculated on 6 replicated measurements of a low (10 µg L^−^^1^ and 100 ng L^−^^1^, for external and matrix-matched, respectively) and a high concentration (1 mg L^−^^1^) within the extent of each calibration, ranged from 2.4 to 6.1%. The matrix effect (ME %), expressing the ratio between the matrix-matched and the external calibration, was 93% and 122% for the first and second set of calibrations, respectively. Thus, the matrix-matched calibrations were used for quantification of exposure concentrations. According to the CPMP/ICH/381/95 guidance document [79], limit of detection and quantification (LOD, LOQ) were determined based on 3.3 and 10 times the standard deviation (SD) of TCE blanks (n = 20) divided by the slope (S) of the respective calibration curve. LOD and LOQ were 18.9 and 57.4 ng L^−1^ for the first, and 9.7 and 29.4 ng L^−1^ for the second matrix-matched calibration. Since not all exposure concentrations were sampled for analysis, the non-sampled concentrations were interpolated using linear regressions of nominal versus measured values for each experiment. Linearities, expressed in *R^2^*, for these regressions ranged from 0.9588 to 0.9999 (cf. Figure S4 for details).

### 2.7. Statistical Analyses

Statistical analyses and illustrations were performed using Rstudio (Version 1.4.1106, RStudio Team [80]). Statistical differences in the responses between the different exposure concentrations for each experiment were analyzed by conducting a non-parametric Kruskal–Wallis test [81] followed by Dunn’s Test [82] for post hoc comparisons using the ‘FSA’ package (Version 0.8.31). Effects are expressed as relative median response including interquartile range (IQR). Assumptions for normal distribution of the data and homogeneity of variances were assessed using the Shapiro–Wilk Test and the Levene’s Test, respectively. Median lethal or effect concentrations that lead to 50% mortality, or 50% of the observed effect in a dosed population [83], were determined as biological endpoints (LC_50_ and EC_50_, respectively). To estimate the LC/ECs, we used a modified R code [84] incorporating generalized linear models with a probit link function in accordance with OECD guidance document No. 54 [85]. To compare LC/ECs, ratio tests [86] were performed using the ‘ecotox’ package (Version 1.4.2). Significance levels were set to α = 0.05 for all statistical tests. 

## 3. Results

### 3.1. Verification of Exposure Concentrations

Test water from the experiments was chemically analyzed via LC-MS analysis to measure the actual exposure concentrations. Apart from the stock solutions, most measured values were below the respective nominal concentration (cf. Table 1). Measured concentrations of the stock solutions, for example, ranged from 6324–8493 µg L^−^^1^ (105–141%) compared to the estimated nominal concentration of 6000 µg L^−^^1^. On the lower end of the tested concentrations, recoveries ranged from 6–92% of 1.3 µg L^−^^1^ nominal concentration. The highest loss of BP3 occurred in the survival experiment with *A. digitifera* larvae with recovered values ranging from 0.6% to 5.7% of the respective nominal concentrations. On the other hand, analytically verified concentrations were closest to those of the nominal ones in the survival experiment with *A. millepora* larvae, ranging from 67–103%. Recovered concentrations were generally lower in samples from the settlement experiments compared to those of the survival experiments in relation to the nominal concentrations; i.e., recovered concentrations in the settlement assays with *L. purpurea* and *T. faulkneri* ranged from 13–29% after 48 h compared to 8–86% in the survival experiments (cf. Table 1). These analytical challenges have implications for further research, which we will discuss below.

### 3.2. Bioassays

For better comparisons between experiments and species, we present the results in nominal concentrations in the following sections with measured concentrations in parentheses. Water quality parameters were within acceptable limits (Table S6) throughout all assays. In all treatment groups and the controls, DO levels ranged from 6.40 to 7.36 mg L^−1^ and from 6.00 to 7.45 mg L^−1^ at the beginning and at the end of the experiments, respectively. Likewise, measured pH ranged from 7.8 to 8.3 at the test start and test end. Detailed results of the bioassays and statistical analyses can be found in Tables S7 and S8–S15, respectively.

#### 3.2.1. Survival Bioassays

**Figure 2:**
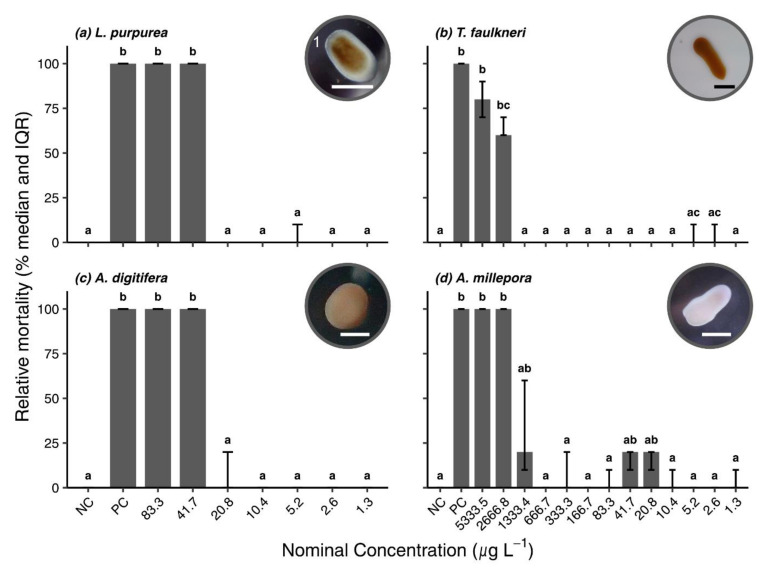
Results of the survival bioassays with (**a**) *Leptastrea purpurea*, (**b**) *Tubastraea faulkneri*, (**c**) *Acropora digitifera* and (**d**) *A. millepora* after 48 h exposure to BP3. Relative median mortality rate and interquartile range (IQR) are shown for each treatment (n = 3). Letters **a**–**c** indicate significance groups obtained from post hoc Dunn’s test, with same letters indicating no significant differences. Pictures showing healthy planula larvae. Scale bar = 500 µm; NC = negative control; PC = positive control; ^1^ Photo courtesy of L. Fiegel.

Survival in the negative controls was 100% in all bioassays after 48 h and thus within the acceptable limits (cf. Section 2.4). All larvae died in the positive controls within 48 h, verifying the suitability of the test system for the anticipated endpoint. Mortality rates were similar for *L. purpurea* and *A. digitifera*, and for *T. faulkneri* and *A. millepora* larvae, respectively, after 48 h exposure to BP3. In fact, 100% mortality occurred at the two highest concentrations for *L. purpurea* and *A. digitifera*, respectively (Figure 2a,c). For both latter species, no significant mortality rates were observed (Dunn’s Test: *p* > 0.05) below a nominal concentration of 41.7 µg L^−1^ (measured 21.6 and 24.9 µg L^−1^, respectively). *T. faulkneri* and *A. millepora* larvae were less susceptible to BP3 exposure with observed mortalities occurring at concentrations about two orders of magnitudes higher compared to *L. purpurea* and *A. digitifera* (Figure 2). Compared to the negative controls, significant effects for *T. faulkneri* only occurred in the two highest concentrations (Dunn’s Test: *p* < 0.05) with 80% (IQR 70–90%) and 60% (IQR 60–70%) mortality at 5333.5 µg L^−1^ (measured 1040 µg L^−1^) and 2666.8 µg L^−1^ (measured 684.4 µg L^−1^), respectively (Figure 2b). Similarly, *A. millepora* mortality was 100% in the two highest treatments, and 20% (IQR 10–60%) in the next lowest one at 1333.4 µg L^−1^ (measured 1289.8 µg L^−1^). Some minor mortality rates were observed in the subsequent dilutions (≤20%), these, however, did not differ from the negative control (Figure 2d; Dunn’s Test: *p* > 0.05).

#### 3.2.2. Settlement Bioassays

**Figure 3:**
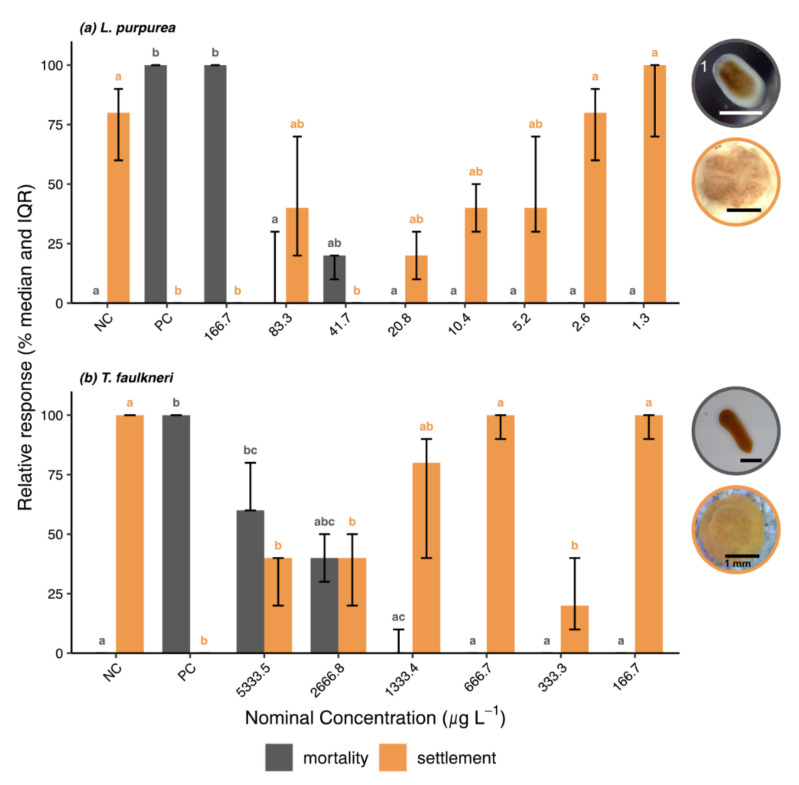
Results of the settlement assays with (**a**) *Leptastrea purpurea* and (**b**) *Tubastraea faulkneri* after 48 h exposure to BP3. Relative median mortality and settlement rate and interquartile range (IQR) are shown for each treatment (n = 3). Letters **a**–**c** indicate significance groups obtained from post hoc Dunn’s test, with same letters indicating no significant differences. Pictures (grey circle): swimming larva; pictures (orange circle): larva undergone settlement; Scale bar = 500 µm unless indicated otherwise. NC = negative control (FSW for survival and FSW and CCA chip for settlement); PC = positive control; ^1^ Photo courtesy of L. Fiegel.

Settlement assays with larvae of the brooding corals *L. purpurea* and *T. faulkneri* were conducted to assess effects on the settlement process in addition to survival effects. Survival after 48 h in the negative controls (FSW + CCA chip) was 100% for both species, while settlement was 100% for *T. faulkneri* and 80% (IQR 60–90%) for *L. purpurea*, and thus, within the defined validity criteria (cf. Section 2.4). Exposure to the positive control resulted in complete mortality in both assays (Figure 3). BP3 exposure significantly affected the survival in the highest concentration in both species (Dunn’s Test: *p*-values < 0.05). Mortality was 100% at 166.7 µg L^−1^ (measured 43.0 µg L^−1^) and 60% (IQR 60–80%) at 5333.5 µg L^−1^ (measured 1201.8 µg L^−1^) for *L. purpurea* and *T. faulkneri*, respectively. Death rates in subsequent lower concentrations were ≤20% for *L. purpurea* and ≤40% for *T. faulkneri*; however, they did not differ significantly from the negative controls (Dunn’s Test: *p*-values > 0.05).

In general, settlement was inhibited at concentrations where mortality occurred, and gradually increased with decreasing mortality and exposure concentrations. In fact, all *L. purpurea* larvae had died before they could settle at the highest test concentration (Figure 3a). Settlement was inhibited thereafter and ranged from 20–100%. Except for the third treatment group (nominal 41.7 µg L^−1^; measured 10.8 µg L^−1^) where settlement was inhibited completely, settlement rates were not significantly different from the negative control (Dunn’s Test: *p*-values > 0.05).

Among the four larval species tested, *T. faulkneri* was the least sensitive species after exposure to BP3 (Figure 3b) with significant mortality observed at the highest test concentration of 5333.5 µg L^−1^ (measured 1201.8 µg L^−1^). BP3 significantly inhibited settlement (40%, IQR 20–50%) down to 2666.8 µg L^−1^ (measured 684.4 µg L^−1^), compared to the negative control (Dunn’s Test: *p*-values ≤ 0.05). At lower concentrations, settlement increased stepwise to the NC level (settlement ≥ 80%, Table S7; Dunn’s Test: *p*-values > 0.05), except for the second lowest concentration (333.3 µg L^−1^; measured 52.2 µg L^−1^) where settlement dropped to 20% (IQR 10–40%) (Dunn’s Test: *p* = 0.027).

### 3.3. Biological Endpoints

**Figure 4:**
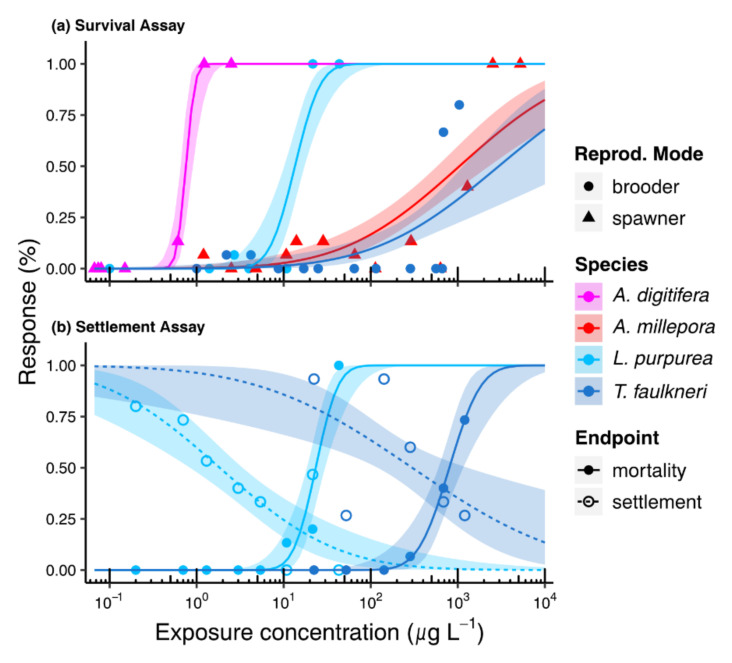
Concentration (measured)—Response (mean, n = 3) relationship of the larval experiments after 48 h BP3 exposure. Probit regressions with shaded areas indicating 95% confidence intervals illustrated for (**a**) survival and (**b**) settlement assays.

The analytically verified exposure concentrations were used to estimate the 50% lethal and effect concentrations (LC/EC_50_) for each assay. Derived toxicities within the survival experiment revealed significant interspecific variations (ratio test: *p*-values ≤ 0.05; Table S17) after BP3 exposure for 48 h (Figure 4a) with LC_50_s ranging from 0.75 µg L^−1^ for *A. digitifera* to 2951.24 µg L^−1^ for *T. faulkneri* (cf. Table 2 and Table S16). Moreover, there were no patterns observable between the reproductive modes (i.e., brooders vs. spawners) in the four tested planulae species. The spawner *A. digitifera* and the brooder *L. purpurea* showed measured LC_50_s in the µg L^−1^ range (i.e., 0.75 µg L^−1^ and 13.47 µg L^−1^, respectively), while larvae of the other spawning and brooding species were much less susceptible to BP3 (ca. 1000 fold increase) with LC_50_s in the mg L^−1^ range (i.e., 1042.31 µg L^−1^ and 2951.24 µg L^−1^, respectively; Figure 4a, Table 2).

Likewise, the derived LC_50_ and EC_50_ values also indicated variations between the two tested brooding species in the settlement assays. *L. purpurea* larvae were more susceptible in the settlement experiment than those of *T. faulkneri*, and settlement was generally affected before survival (Figure 4b, Table 2). In fact, the LC_50_ of *T. faulkneri* (799.84 µg L^−1^) was 34-fold larger than that of *L. purpurea,* which had a LC_50_ of 23.35 µg L^−1^ (ratio test: *p*-value = 0, Table S18). Similarly, the settlement process of *L. purpurea* planulae was affected at an EC_50_ (1.84 µg L^−1^) about two orders of magnitude lower compared to *T. faulkneri* (298.92 µg L^−1^; ratio test: *p*-value = 3.9 × 10^−5^, Table S19). For both species, the EC_50_s were significantly lower than the LC_50_s (Table 2; ratio test: *p*-values ≤ 0.05, Table S20).

Intraspecific variations were observed in the toxic responses between the survival and settlement bioassays (Figure 4, Table 2). For *L. purpurea* planulae, the LC_50_ was about 2-fold lower in the survival experiment compared to the settlement experiment (i.e., 13.5 µg L^−1^ vs. 23.4 µg L^−1^; ratio test: *p*-value = 4.6 × 10^−5^, Table S21). In contrary, *T. faulkneri* planulae showed an almost 4-fold lower LC_50_ in the settlement compared to the survival assay (i.e., 799.84 µg L^−1^ vs. 2951.24 µg L^−1^; ratio test: *p*-value = 2.6 × 10^−11^, Table S21). Implications of these observations will be discussed in the following sections.

## 4. Discussion

**Figure 5:**
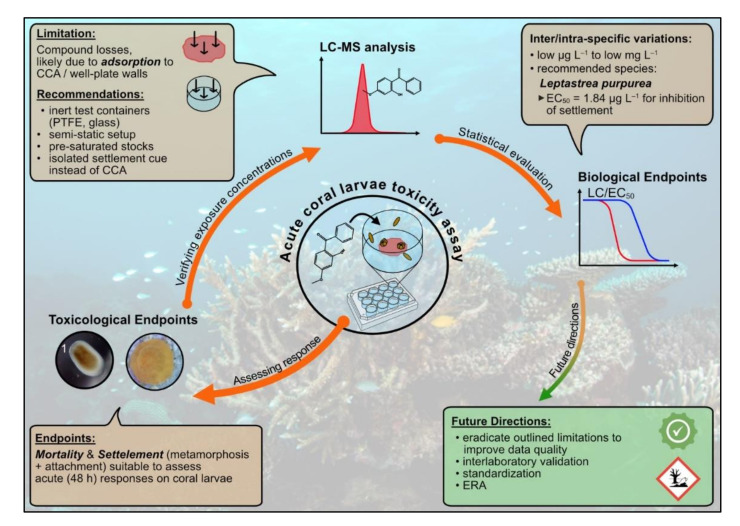
Schematic summary of the here performed experiments and data analyses. ^1^ Photo courtesy of L. Fiegel.

We conducted acute toxicity assays exposing planula larvae of four scleractinian corals to the UV filter BP3 aiming to contribute to the development of a standardized toxicity test for corals. We found that the test system is suitable in principle to assess both mortality and inhibition of settlement as acute toxicity endpoints for planula larvae of various scleractinian coral species. Results indicated species-specific variations in biological endpoints (i.e., LC/EC_50_) based on analytically verified exposure concentrations. The limitations and implications of these results are discussed in the following sections and a summary of this discussion is provided in Figure 5.

### 4.1. Limitations and Implications for the Development of a Standard Toxicity Test for Corals

#### 4.1.1. Assay Performance and Choice of Toxicological Endpoints

We used a well plate-based setup similar to previously presented protocols [72,73,84] for static exposure assays. In this paper, we presented the proposed test exemplified using the UV filter BP3. In addition, we tested the suitability of the experimental setup with other UV filters (i.e., octocrylene or OCR, ethylhexyl-methoxycinnamate or EHMC) and other lipophilic compounds (e.g., cinnamic acid benzyl ester, anethole), as well as with further potential positive controls such as diuron and copper for *L. purpurea* and *A. digitifera* planulae (cf. SI S3, Figures S6 and S7, Tables S23 and S24). Based on the results of this study (including the additional results in SI S3), and in agreement with previous coral larvae toxicity studies [67,87], we consider mortality and inhibition of settlement to be useful endpoints when investigating acute toxic effects from chemical exposure to coral larvae. The survival of coral planulae depends on the successful recruitment, which in turn is determined by their ability to identify a suitable substrate to settle on [10,53,86]. In other words, a substance that does not affect survival but prevents the planula larvae from successful settlement will ultimately have the same effect on the reef as larval mortality [54]. Although inhibition of settlement is a sublethal response, it is, due to the direct link to larval survival, in our view a population relevant endpoint. We generally observed the inhibition of settlement at lower concentrations than mortality, and thus, investigating survival alone may underestimate toxicities. This was particularly the case for coumarin, which showed ≤20% larval mortality at nominal 88.9 mg L^−1^, but effectively inhibited settlement in *A. digitifera* planulae at nominal concentrations ≥ 2.8 µg L^−1^ (Figure S7). Downs et al. [36] studied mortality and also deformation as sublethal endpoint in *Stylophora pistillata* planulae after exposure to BP3. However, the ecological relevance of sublethal effects of such morphological changes in planulae are yet to be demonstrated [52]. Compared to other acute coral larvae toxicity studies [67,87,88], which used a period of 24 h to investigate settlement in *A. tenuis* planulae, we chose a test duration of 48 h. This was due to the circumstance that for the species we tested, a 24 h period was insufficient to achieve ≥70% settlement (metamorphosis and attachment) within the negative control (data not shown), which is in agreement with previous settlement studies [73,89,90]. Thus, a test duration of 48 h is considered appropriate for the assessment of both investigated endpoints.

While most UV filter coral toxicity studies so far have not run positive controls with their experiments [49,50,52], we have included TBTO as a reference toxicant. However, we recommend using alternative reference toxicants as positive controls in future studies so that settlement inhibition can be induced besides mortality, and also due to TBTO’s toxicity towards humans [71]. A recent study by Conway et al. [91] used copper and diuron to induce bleaching and mortality in adult *Galaxea fascicularis* corals, respectively.

#### 4.1.2. Considerations for Settlement as an Endpoint

CCA chips effectively induced settlement at rates above 70% in the negative controls for the tested species (*L. purpurea* and *T. faulkneri*) in the settlement assays. Similar results have been obtained in previous studies using CCA or associated extracts to induce settlement, e.g., [67,73]. However, live CCA may be problematic for the utilization as a settlement inducer in standardized toxicity tests for several reasons. CCA covered substrates are typically very porous and heterogenous structures that can introduce undesirable organisms such as ciliates or other microorganisms into the test system [73], potentially affecting the water chemistry and interfering with the target compound. Especially lipophilic substances such as UV filters could be adsorbed to CCA substrate, which may reduce the substance concentration in the test media available to the test organism [75,92]. CCAs are also known to compete with colonizers (i.e., coral larvae or other epibiont) through defense mechanisms such as mucus secretion and epithelial sloughing, potentially reducing recruit survival [93,94]; the latter has previously been observed in *L. purpurea* [73]. We also observed possible interactions between CCA and *L. purpurea* planula while conducting bioassays in Guam. Specifically, after replacing our CCA stock with freshly collected ones from the reef, planulae turned pink (see Figure S5) and subsequently died within 48 h. We did not further investigate this observation as it was beyond the scope of this study. The phenomenon fully subsided after about one week of acclimation in the UOGML flow-through system. Due to the described potential complications using live CCA in settlement assays, we suggest the consideration of alternative settlement cues for the development of a standardized coral toxicity test. Petersen et al. [89] recently identified the morphogenic red pigment cycloprodigiosin, produced by the CCA-associated marine bacterium *Pseudoalteromonas rubra* #1783 [90], as a potent chemical cue to induce full settlement (metamorphosis and attachment) in *L. purpurea* planula larvae at reliably high success rates of nearly 90% after 48 h in combination with an alternating 12 h dark and light cycle. Applying such an isolated chemical cue would be ideal in toxicity assays, as it may counteract secondary effects, as well as compound loss caused by interactions of CCA with the media and/or target compound, and to assure optimized reliability and replicability. Since larvae are very difficult to localize on the heterogenous surfaces of CCAs, we quantified responses only after 48 h to avoid disturbances during the settlement process. The use of isolated cues rather than live CCA substrate would therefore improve identification of planulae and quantification of responses and would also enable temporal data generation. However, for the utilization of a settlement agent such as cycloprodigiosin in a standardized coral bioassay, its suitability as a reliable cue for other representative coral species needs to be further evaluated.

#### 4.1.3. Coral Recruits as Another Possible Early Life Stage Endpoint

Although this study focused on toxicities towards coral larvae, we conducted additional experiments on two-month-old post-settlement recruits of *A. digitifera* as the most sensitive species within the larvae tests to assess effects on another sensitive early life stage, which has so far been ignored in previous UV filter coral toxicity studies [49]. Using the same test setup as in the larval assays, we investigated the suitability of the endpoints tissue necrosis, polyp reactivity and mortality after 48 h static BP3 exposure and observed an increase in polyp reactivity, as tissue necrosis and mortality decreased (cf. SI S4, Figures S8–S10, Tables S25–S27). We further observed that polyps were often extended, yet non-responsive, potentially indicating a stress response [95,96]. The practicability of assessing polyp reactivity in corals with smaller sized polyps, such as *Porites* spp. or *Montipora* spp., requires further investigation. Moreover, the suitability of sublethal effects such as polyp reactivity in coral toxicity testing has recently been questioned since its role in coral health is unclear [50,91]. A period of 72 h was used for the determination of effects on *A. microphthalma* recruits from exposure to the antifoulant tributyltin [97], and effects of diuron on *A. millepora* and *P. damicornis* recruits were assessed after 96 h exposure by Negri et al. [60]. Therefore, longer test durations of 96 h may be more appropriate for the assessment of acute toxic effects on coral recruits. We suggest that the larval stage is more suitable to represent the early life stage of corals in toxicity tests than recruits based on our results (i.e., less sensitive compared to the larval stage), and since recruit generation is technically challenging and labor intense, requiring extensive infrastructure and highly qualified personnel.

#### 4.1.4. Analytical Challenges in Small Water Volumes

Analytical recovery of lipophilic substances such as BP3 remains a challenge in small volume static test setups. The observed low recoveries, especially in the lower concentration treatments, were likely caused by adsorption to the polystyrene well plates and/or to the CCAs, effectively removing the lipophilic compound from the test water and thus reducing the available exposure concentration for the larvae [75,98]. To improve analytical recoveries, the use of well plates consisting of more inert materials such as glass ware [99,100] or even Teflon (PTFE)-coated materials [35,36,75], instead of plastic well plates, should be considered in further experiments. A further option to enable more consistent exposure concentrations might be the modification from a static to a semi-static setup [75]. Although the analytical measurements were considered as preliminary and further research is needed to improve recovery rates in such saltwater-based tests with low water volumes, it is well known that especially for highly lipophilic substances such as UV filters, a constant water concentration is a major challenge [101]. The most substantial compound losses were observed in the treatment samples of the *A. digitifera* survival experiment (cf. Table 1). It is unclear if this was due to larger sorption effects, greater uptake by larvae, or if the samples itself or the extractions were compromised. As a consequence, derived toxicity thresholds for this species based on measured treatment concentrations might be inaccurate and should be taken with caution.

Due to their high lipophilicity and low water solubility, UV filters are difficult to test substances [75]. For such substances, the use of solvents may be required to maintain a constant concentration in the water. The use of solvents is generally not recommended in toxicity tests, however, if unavoidable, a solvent concentration of 0.01% (100 µL L^−1^) should not be exceeded and a solvent control is required in addition to a negative control (test medium), as recommended in OECD guidance document No. 23 [75]. All to date published toxicity studies assessing effects of organic UV filters on corals used carrier solvents. Some of the studies used methanol; three of which [40,41,91] at 0.01% and one [43] at 6.7 × 10^−3^%. Danovaro et al. [47] used 3.3 × 10^−3^% propylene glycol; however, a solvent control was reported only for experiments conducted in one of several study locations. The remaining studies utilized dimethyl sulfoxide (DMSO), even though it is known for its interactions with biological membranes and thus enhanced the uptake of the test substance [102,103]; an effect which cannot be managed by solvent control as further discussed by Mitchelmore et al. [50]. Employed DMSO levels ranged from very low (5 × 10^−4^%; [36]) to very high (0.25%; [42,48]); the latter by far exceeding the limit set in OECD No. 23 [75], and additionally missing negative controls (only solvent controls were included). Wijgerde et al. [46] used 0.01% DMSO; however, as only a solvent control was included (no negative medium control), observed effects in that control (33% mortality) made the study results unreliable [50,52]. Despite that, even if the solvent concentration is well below the maximum recommended level (0.01%; [75]), secondary effects cannot be ruled out since there is a paucity of studies assessing solvent effects on corals. We observed morphological alterations in adult reef-building corals exposed to even lower solvent concentrations than those reported in the studies above which may impact coral health and might not be addressable by a solvent control alone (unpublished data [104]). Therefore, we cannot recommend using solvents in any standardized test system and decided not to do so in this study; instead, we produced a saturated stock solution by the means of the direct addition of FSW to the UV filter in a glass bottle and subsequent stirring over a time period of 24 h (cf. Section 2.3). Measured concentrations on the resulting BP3 stock solutions ranged from 105% to 141% compared to the known water solubility limit in pure water (Table 1), thus verifying the suitability of the method. The preparation of the test media may be further improved by pre-saturating the stock solution container using a solid–liquid saturator system [75,105].

### 4.2. Toxicity Thresholds

Derivation of the lethal and effect concentrations (LC/EC_50_) revealed interspecific variations in the toxic responses towards BP3 exposure, but also intraspecific differences between the distinct bioassay types. Toxicities varied from the low µg L^−1^ to low mg L^−1^ range. The most sensitive larvae towards BP3 in our study were of the broadcast spawner *A. digitifera* with a LC_50_ (0.75 µg L^−1^) about four orders of magnitude lower than the least affected planulae of the brooding species *T. faulkneri* (LC_50_ = 2951.24 µg L^−1^). The second most susceptible species were brooding larvae of *L. purpurea* with the observed inhibition of settlement (EC_50_ = 1.84 µg L^−1^) in roughly the same order of magnitude compared to the LC_50_ of *A. digitifera*. We also found intraspecific variations in the lethal responses when comparing the LC_50_ values of the same species between the survival and the settlement experiment. While the specific reasons cannot be determined without further investigations, we suspect CCA interactions to be responsible; either through adsorption of the compound to CCA (in the case of *L. purpurea* as LC_50_ in the settlement experiment 2-fold higher than in the survival experiment), or through secondary effects caused by CCA and related microorganisms (in the case of *T. faulkneri* with lower LC_50_ in the settlement assay). As discussed before (cf. Section 4.1.2), the use of chemical settlement triggers [73,89] has the potential to avoid such interactions and should therefore be further investigated.

Despite the expectations, we observed contrasting intragenus lethal responses between the two *Acropora* species, with a three orders of magnitude higher LC_50_ in *A. millepora* (the difference is reduced by about 50% when comparing the nominal values; cf. Table S16). While the reasons for these differences are unclear, intragenus variations were also apparent in other studies. For example, *A. millepora* larvae were more sensitive (ca. 6-fold) to petroleum hydrocarbons [106] than larvae of *A. tenuis* [64]; however, differences were less pronounced compared to this study. Additional experiments are required to further investigate the observed differences within the *Acropora* genus and to draw conclusions.

Quantitative comparisons with previous studies are difficult due to methodological and analytical inconsistencies, and due to the lack of analytical verification of exposure concentration in various previous studies as discussed in recent literature [49,50,51,52]. Up to this date, only two other studies performed toxicity assays with UV filters on coral larvae. Downs et al. [36] reported the LC_50_ for *S. pistallata* planulae to be 139 µg L^−^^1^ after 24 h exposure to BP3 in light conditions; however, the study lacks analytical verification of exposure concentrations. He et al. [40] on the other hand, reported lower toxicities with mortality and inhibition of settlement from BP3 exposure in *S. caliendrum* planulae at LC/EC_50_s > 1000 µg L^−^^1^. However, the exposure duration in the latter study was 14 d for coral larvae, indicating a chronic study, while the derivation of LC/EC_50_s and the risk assessment suggest acute test conditions, causing some confusion over the nature of the performed toxicity assay [50].

Other early life stages were not included in any of the previous UV filter toxicity studies. BP3 exposure of *A. digitifera* post-settlement recruits resulted in an LC_50_ of 218.64 µg L^−^^1^ and an EC_50_ of 132.32 µg L^−^^1^ for tissue necrosis after 48 h (cf. SI S4), indicating higher resistance towards chemical exposure compared to the larval stage of the same species and of *L. purpurea*. On the other hand, they were less tolerant than larvae of *A. millepora* and *T. faulkneri*. However, we only tested recruits of one species, thus, further acute experiments including various early, as well as adult life stages of a wider range of representative species, are needed for conclusive results.

### 4.3. Future Directions

Due to the remaining analytical limitations, and since the purpose of this study was to investigate the suitability of this test method for further standardization within the ISO and/or OECD framework, the results presented should not be used for the purpose of a marine environmental risk assessment. Instead, the proposed test design should be used as a baseline and further improved using the recommendations outlined, and interlaboratory validation should follow for the development of a standard coral toxicity test. This would ensure sufficient reproducibility and enable the comparison of results for several substances of concern and a variety of coral species, and allow for higher tier ERA. This, in turn, would enable policymakers to make scientifically sound decisions to minimize local stressors. Based on our results and their implications discussed above, we recommend the consideration of *L. purpurea* as a representative species for the development of an early life stage standard acute toxicity test for corals. Although the brooding reproductive strategy represents only a mere 20% of known coral species, they have the advantage of frequent larval supply compared to broadcast spawners, which supply offspring on an annual basis, thereby limiting research capacities [107,108,109]. In fact, *L. purpurea* has been shown to produce competent planulae on a daily basis over prolonged periods, making this species a well-suited organism [72] for ecotoxicological experiments where a regular supply of larvae is critical. However, efforts should be made to find additional suitable, widely available and easy to culture candidate species as representatives for scleractinian corals.

## 5. Conclusions

Here, we proposed a robust bioassay to assess the acute responses of planula larvae of four scleractinian coral species to anthropogenic compounds, exemplified on the commonly used UV filter BP3. We found that mortality and settlement (metamorphosis and attachment) are useful endpoints in coral larvae toxicity testing. Interspecific variations in toxicity thresholds based on measured exposure concentrations for the four tested species were observed, ranging from low µg L^−1^ to low mg L^−1^. Settlement inhibition for larvae obtained from the brooding coral *L. purpurea* occurred at an EC_50_ of 1.84 µg L^−1^. The results of the BP3 experiments revealed that the test system with coral larvae, the test duration of 48 h and the investigated endpoints, settlement and mortality, are suitable for further validation and international standardization within the ISO and/or OECD framework. However, the choice of relevant coral species applicable for an interlaboratory-based evaluation, as well as the analytical challenges of the highly lipophilic substances tested within this small-volume setup, remain. Therefore, further research is needed to elaborate on the latter aspects and also to include a broader range of substances displaying various physicochemical properties (i.e., lipophilicity, water solubility).

## Figures and Tables

**Table 1:** Analytical results. Measured concentrations after 48 h presented for each species and experiment compared to nominal concentrations and recoveries.

Nominal Concentration ^1^ (µg L^−1^)	Measured Concentration (µg L^−1^)						Recovery ^2^ (%)
	*L. purpurea*		*T. faulkneri*		*A. millepora*	*A. digitifera*	
	Survival	Settlement	Survival	Settlement	Survival	Survival	
6000 (Stock)	8474.3	6475.5	6848.1	6848.1	6323.7	n.a.	105–141
5333.5	n.t.	n.t.	1040.0	1201.8	5219.4	n.t.	20–98
2668.8	n.t.	n.t.	684.4	684.4	2522.7	n.t.	26–95
1333.4	n.t.	n.t.	661.0 *	284.2 *	1289.8	n.t.	21–97
666.7	n.t.	n.t.	561.6	142.1 *	631.9	n.t.	21–95
333.3	n.t.	n.t.	285.2	52.2	290.3	n.t.	16–87
166.7	n.t.	43.0	114.7	22.3	113.0	n.t.	13–69
83.3	43.5	21.5 *	64.7	n.t.	65.5	2.5	3–79
41.7	21.6 *	10.8 *	24.9 *	n.t.	28.4	1.2 *	3–69
20.8	10.8 *	5.4 *	17.0	n.t.	14.0	0.6 *	3–82
10.4	4.0	3.0	8.7*	n.t.	10.7	0.067	0.6–103
5.2	2.7 *	1.3*	4.2	n.t.	4.8	0.150 *	3–92
2.6	1.4 *	0.7 *	2.2 *	n.t.	2.5 *	0.080 *	3–97
1.3	0.1	0.2	1.0	n.t.	1.2	0.074	6–92
**Recovery ^3^ (%)**	8–54	15–29	20–86	13–26	67–103	0.6–5.7	0.6–103

* Interpolated values based on linear regressions of nominal vs. measured concentrations (cf. Section 2.6.3). **^1^** estimated based on the maximum water solubility (cf. Section 2.3); **^2^** percent recovery range in relation to respective nominal value per concentration step; **^3^** percent recovery in relation to corresponding nominal concentrations per experiment, excluding stock solution. n.a. = sample not available; n.t. = concentration not tested.

**Table 2:** Biological endpoints after 48 h of BP3 exposure. Measured LC/EC_50_ values in µg L^−1^ derived from probit regression analysis including lower and upper 95% confidence intervals in brackets shown for each species and experiment. Extended table including nominal toxicity thresholds can be found in Table S16.

	Survival Assay	Settlement Assay	
Species	LC_50_	LC_50_	EC_50_
*L. purpurea*	13.47 [10.58, 17.14]	23.35 [18.85, 28.93]	1.84 [0.97, 3.47]
*T. faulkneri*	2951.24 [813.63, 10,705]	799.84 [603.80, 1059.54]	298.92 [122.40, 730.00]
*A. millepora*	1042.31 [543.82, 1997.75]	-	-
*A. digitifera*	0.75 [0.66, 0.87]	-	-

## Data Availability

The data presented in this study are openly available in FigShare at figshare.com: 10.6084/m9.figshare.17048549 (accessed on 19 November 2021).

## Supplementary Materials

The following supporting information can be downloaded at: https://www.mdpi.com/article/10.3390/toxics10050244/s1, Figure S1: Extraction efficiency; Figure S2: Extracted ion chromatogram (EIC) of BP3; Figure S3: Calibration curves for quantification of analytes; Figure S4: Linear regressions for interpolation of non-sampled exposure concentrations; Figure S5: CCA induced stress response in planula larvae of *L. purpurea*; Figure S6: Results of additional larval stage settlement assays with *L. purpurea*; Figure S7: Results of additional larval stage settlement assays with *A. digitifera*; Figure S8: Experimental setup of the bioassays including recruits; Figure S9: Results of the bioassay with *Acropora digitifera* recruits after 48 h BP3 exposure; Figure S10: Concentration (measured)—Response relationship of the recruit experiment for *A. digitifera* recruits after 48 h BP3 exposure. Probit regressions with shaded areas indicating 95% confidence intervals illustrated for three endpoints;Table S1: Chemical compounds used in acute larval assays, and their relevant physiochemical properties; Table S2: Overview of conditions for the husbandry of adult corals and reproduction details; Table S3: Overview of experimental conditions for bioassays; Table S4: Nominal concentrations of stock solutions prepared for bioassays; Table S5: Statistical results for vortex time assay; Table S6: Water quality parameters at the start of the experiments and at the end after 48 h; Table S7: Results of larval toxicity bioassays; Table S8: Detailed results of the statistical analyses for the Survival assay with *L. purpurea*; Table S9: Detailed results of the statistical analyses for the Survival assay with *A. millepora*; Table S10: Detailed results of the statistical analyses for the Survival assay with *A. digitifera*; Table S11: Detailed results of the statistical analyses for the Survival assay with *T. faulkneri*; Table S12: Detailed results of the statistical analysis for the endpoint ‘mortality’ in the Settlement assay with *L. purpurea*; Table S13: Detailed results of the statistical analysis for the endpoint ‘settlement’ in the Settlement assay with *L. purpurea*; Table S14: Detailed results of the statistical analysis for the endpoint ‘mortality’ in the Settlement assay with *T. faulkneri*; Table S15: Detailed results of the statistical analysis for the endpoint ‘settlement’ in the Settlement assay with *T. faulkneri*; Table S16: Biological endpoints after 48 h of BP3 exposure; Table S17: Statistical comparison of LC_50s_ between species in the Survival experiment performed by ratio test; Table S18: Statistical comparison of LC_50s_ between species in the Settlement experiment performed by ratio test; Table S19: Statistical comparison of EC_50s_ between species in the Settlement experiment performed by ratio test; Table S20: Statistical comparison of LC_50_ vs. EC_50_ values between species in the Settlement experiment performed by ratio test; Table S21: Statistical comparison of LC_50s_ between the same species in the Survival vs. the Settlement experiment performed by ratio test; Table S22: Statistical comparison of the most sensitive biological endpoints in the Survival vs. the Settlement experiment performed by ratio test; Table S23: Chemical compounds tested in additional ecotoxicological experiments and their relevant physiochemical properties; Table S24: Nominal concentrations of saturated stock solutions used for serial dilutions in ecotoxicological experiments; Table S25: Overview of experimental conditions for recruit bioassay; Table S26: Results of toxicity bioassay with *A. digitifera* recruits; Table S27: Multiple comparisons for the recruit bioassay.
