# Supplementary material for: Towards the Development of Standardized Bioassays for Corals: Acute Toxicity of the UV Filter Benzophenone-3 to Scleractinian Coral Larvae
Source: Toxics. 2022 May 10;10(5):244. doi: 10.3390/toxics10050244 (PMC9146638; doi:10.3390/toxics10050244)
Supplement: Supplementary file 1 [file toxics-10-00244-s001.zip › toxics-1716983-supplementary.pdf]

toxics

Supplemenatry Materials: Towards the Development of Standardized Bioassays for Corals: Acute Toxicity of the UV Filter Benzophenone-3 to Scleractinian Coral Larvae

Ingo B. Miller, Mareen Moeller, Matthias Y. Kellermann, Samuel Nietzer, Valentina Di Mauro, Elham Kamyab, Sascha Pawlowski, Mechtild Petersen-Thiery and Peter J. Schupp

S1: Methods

Table S1. Chemical compounds used in acute larval assays, and their relevant physiochemical properties.

Compound	Formula	CAS No.	Structure	Water solubility	Log K_{ow}	Density (g cm^{-3})	Comments	Acquired from
Benzophenone-3; 98% [BP3]	$C_{14}H_{12}O_3$	131-57-7	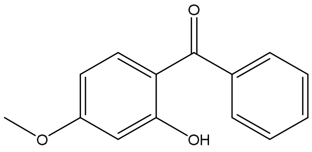	^{a}6 mg L^{-1} (25 °C)	^{a}3.45 (40 °C)	^{a}1.43 (20 °C)	UVA, UVB	Sigma Aldrich Chemistry (Taufkirchen, Germany)
Bis(tri-n-butyltin) oxide; 97% [TBTO]	$C_{24}H_{54}OSn_2$	56-35-9	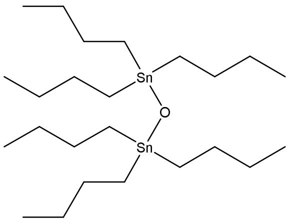	^{a}71.2 mg L^{-1} (20 °C)	^{a}2.2	^{a}1.17 (28 °C)		abcr GmbH, Karlsruhe, Germany

Sources: ^{a} ChemSpider [1]; ^{b}(obtained from Sigma Aldrich safety data sheets); ^{c} ChemicalBook [2]; ^{d} ECHA REACH registration dosier [3].

Table S2. Overview of conditions for the husbandry of adult corals and reproduction details.

**Species**	*Acropora digitifera*	*Leptastrea purpurea*	*Acropora millepora*	*Tubastraea faulkneri*
**Reproduction mode**	Broadcast spawning	Brooding	Broadcast spawning	Brooding
**Origin**	Reef crest Pago Bay, Guam<br>13°25'35"N, 144°47'47"E	Reef flat Luminao Reef, Guam<br>13°27'56"N, 144°38'48"E	Northern Great Barrier Reef, Australia	Indonesia
**Permit**	Special License for the Collection of Coral' issued to the UOGML by the Guam Department of Agriculture (DAWR)	Special License for the Collection of Coral' issued to the UOGML by the Guam Department of Agriculture (DAWR)	CITES permit No: 20NL284183/11	CITES permit No: 20NL288025/11
**Husbandry Location**	UOGML, Guam	UOGML, Guam	ICBM, Germany	ICBM, Germany

**No of adult colonies**	8	100	8	6
**Colony size (approximate diameter)**	25 cm	5–10 cm	8–10 cm	10 cm
**Culture water**	Natural reef water	Natural reef water	Artificial seawater: Tropic Marin Pro-Reef salt, dissolved in reverse osmosis water at 39 g L^{-1}	Artificial seawater: Tropic Marin Pro-Reef salt, dissolved in reverse osmosis water at 39 g L^{-1}
**System**	Outdoor open natural seawater flow-through system	Outdoor open natural seawater flow-through system	Closed ex situ recirculating system with artificial seawater	Closed ex situ recirculating system with artificial seawater
**System volume**	4000 L	400 L	1700 L	6500 L
**Tank volume**	4000 L	400 L	600 L	250 L
T (°C, mean ± SD)	30.2 ± 0.5 [1]	30.2 ± 0.5 [1]	26–27	26–27
pH	7.7–8.2	7.7–8.2	7.0–8.3	7.9–8.3
DO (mg L^{-1})	6.5–7.5	6.5–7.5	6.5–7.5	6.5–7.5
S	35	35	~34	~34
Alkalinity	Natural reef water	Natural reef water	7–7.5 °dKH	7–7.5 °dKH
Phosphate	Natural reef water	Natural reef water	0.05–0.15 mg L^{-1}	0.05–0.15 mg L^{-1}
**Light**	Natural	Natural	Artificial (LED)	Artificial (LED)
Diurnal cycle	~12h day / 12 h night	~12h day / 12 h night	Synchronized with Northern GBR	~12h day / 12 h night
Light intensity (day: 6am – 6 pm)	4975 ± 9792 LUX range: 11–132268^{-1} [~90 µmol m^{-2} s^{-1}]	4975 ± 9792 LUX range: 11–132268^{-1} [~90 µmol m^{-2} s^{-1}]	~5400 LUX [~100 µmol m^{-2} s^{-1}]	~5400 LUX [~100 µmol m^{-2} s^{-1}]
**Lunar cycle**	Natural	Natural	Synchronized with GBR spawning	None
**Reproduction**	July 2019, spawning	August–October 2019, brooding	December 2020, spawning	February 2021, brooding
**Acclimatization before spawning/larval collection**	7–10 days	7 days	7–10 days	7 days

UOGML = University of Guam Marine Laboratory; ICBM = Institute for Chemistry and Biology of the Marine Environment, University of Oldenburg, Wilhemshaven, Germany.
[1]HOBO Pendant UA-002-64, Onset Computer Corporation, Bourne, MA, USA.

Table S3. Overview of experimental conditions for bioassays.

Experiment	Larval Survival				Larval Settlement	
**Species:**	***A. digitifera***	***L. purpurea***	***A. millepora***	***T. faulkneri***	***L. purpurea***	***T. faulkneri***
**Well plate:**	12-well cell culture plate; Polystyrene (Product No. 92412; TPP Techno Plastic Products AG, Switzerland)					
**Water:**						
- Origin (cf. Table S1.1 for details)	Natural seawater	Natural seawater	Artificial seawater	Artificial seawater	Natural seawater	Artificial seawater
- Pre-treatment for stock solutions and bioassays	0.22 µm filter sterilized	0.22 µm filter sterilized	0.22 µm filter sterilized	0.22 µm filter sterilized	0.22 µm filter sterilized	0.22 µm filter sterilized
Abbreviation	FSW	FSW	FAW	FAW	FSW	FAW
**Test Media:**						
- Target chemical	BP3	BP3	BP3	BP3	BP3	BP3
- Negative (media) control	FSW	FSW	FAW	FAW	FSW + CCA[1]	FAW + CCA[1]
- Positive control	1.2 mg TBTO L^{-1} FSW	1.2 mg TBTO L^{-1} FSW	1.2 mg TBTO L^{-1} FAW	1.2 mg TBTO L^{-1} FAW	1.2 mg TBTO L^{-1} FSW	1.2 mg TBTO L^{-1} FAW
- Volume/well	4.5 mL	4.5 mL	4.5 mL	4.5 mL	4.5 mL	4.5 mL
**Settlement inducer:**	-	-	-	-	CCA[1]	CCA[1]
**Replicates:**	3	3	3	3	3	3
**Pseudo-replicates:**	5 larvae/well	5 larvae/well	5 larvae/well	5 larvae/well	5 larvae + CCA (~0.5 cm^3) / well	5 larvae + CCA (~0.5 cm^3) / well
**Exposure concentrations (nominal):**	6 (2-fold serial dilution ranging from 83.3–1.3 µg/L) + controls	6 (2-fold serial dilution ranging from 83.3–1.3 µg/L) + controls	13 (2-fold serial dilution ranging from 5333.5–1.3 µg/L) + controls	13 (2-fold serial dilution ranging from 5333.5–1.3 µg/L) + controls	8 (2-fold serial dilution ranging from 166.7–1.3 µg/L) + controls	6 (2-fold serial dilution ranging from 5333.5–166.7 µg/L) + controls
**Incubation conditions:**						
- Duration:	48 h	48 h	48 h	48 h	48 h	48 h

- Light source:	Artificial (white light source)	Artificial (white light source)	Artificial (LED)	Artificial (LED)	Artificial (LED)	Artificial (LED)
- Light cycle: day/night	12h/12h	12h/12h	12h/12h	12h/12h	12h/12h	12h/12h
- Light intensity:	~4300 LUX [~80 μmol m^{-2} s^{-1}]	~4300 LUX [~80 μmol m^{-2} s^{-1}]	4300 LUX [80 μmol m^{-2} s^{-1}]	4300 LUX [80 μmol m^{-2} s^{-1}]	4300 LUX [80 μmol m^{-2} s^{-1}]	4300 LUX [80 μmol m^{-2} s^{-1}]
- Temperature:	29–30	29–30	26–27	26–27	29–30	26–27
**Toxicological endpoints:**	Mortality	Mortality	Mortality	Mortality	- Settlement<br>- Mortality	- Settlement<br>- Mortality

[1]CCA = crustose coralline algae (*Hydrolithon reinboldii*) originated from Luminao Reef, Guam (13°27'56"N, 144°38'48"E).

Table S4. Nominal concentrations of stock solutions prepared for bioassays.

**Compound**	**Abbreviation**	**Stock solution ($mg\ L^{-1}$ FSW)**	**Saturated stock solution [adjusted to known water solubility] ($mg\ L^{-1}$ FSW)**
benzophenone-3	BP3	12	6
Bis(tri-n-butyltin) oxide	TBTO	140	70

Extraction Efficiency

Zhang and Lee [4] reported a vortex time of 3 min to achieve the best dispersion result. However, since the aforementioned study used different matrices such as ultrapure water and river water compared to our setup with seawater samples, we conducted a pre-experiment to determine the most efficient vortex duration. Therefore, artificial seawater samples (Tropic Marine salt dissolved in omnipure water, 35 g L^{-1}) were filter sterilized with 0.22 μm Nitrocellulose filter (Ø 47 mm, GVS North America, Sanford, ME, USA) and spiked with 10 μg L^{-1} BP3 dissolved in EtOH. Three replicates were prepared for four different vortex durations, respectively. Each sample was extracted by vortexing at 2500 rpm for 0.5, 1, 2 and 3 min. The results demonstrated significant differences in peak areas in relation to vortex time (ANOVA, $F(3, 8) = 6.1171$, $p = 0.0182$). Extraction efficiency increased significantly from 0.5 to 1 min vortex duration (Tukey test, $p = 0.0236$) followed by a decreasing response thereafter (Figure S1, Table S5). Thus, a vortex time of 1 min was chosen as the most efficient duration for this test setup.

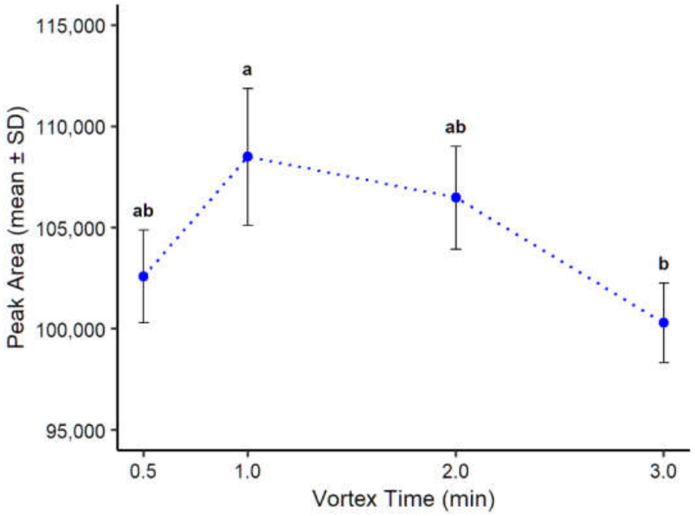

Figure S1. Extraction efficiency. Extraction efficiency in relation to vortex duration for a sample with a BP3 concentration of 10 µg L^{-1}, n = 3 replicates each. Data shown in mean values ± SD. Letters indicate significance groups obtained from post hoc Tukey test following ANOVA, where different letters indicate significant differences.

Table S5. Statistical results for vortex time assay.

Statistical Test	Comparison	*p*-value
Shapiro–Wilk normality Test	-	0.3267
Bartlett test of homogeneity of variances	-	0.9089
Analysis of Variances (ANOVA)	-	0.01818
Tukey post hoc comparisons	0.5–1	0.02361
	0.5–2	0.10402
	0.5–3	0.31084
	1–2	0.36767
	1–3	0.00474
	2–3	0.01942

Chemical Analytics

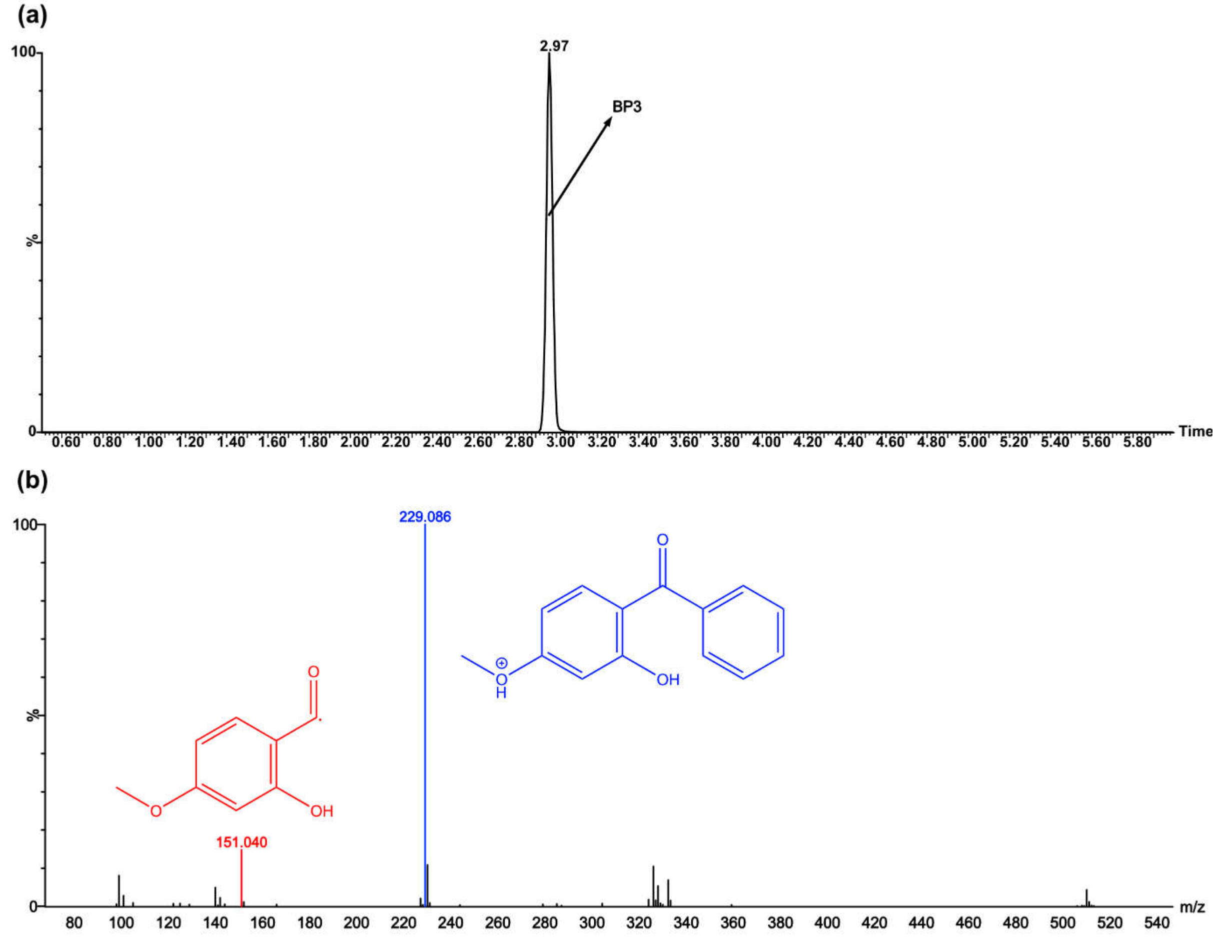

Figure S2. Extracted ion chromatogram (EIC) of BP3. EIC of Σ of *m*/*z* 229.086 and *m*/*z* 151.040 (**a**), and mass spectra (**b**) at a retention time of 2.97 min. Protonated parent molecule at *m*/*z* 229.086 $[M+H]^+$ and in-source fragment ion of BP3 at *m*/*z* 151.040 were both used for peak integration. .

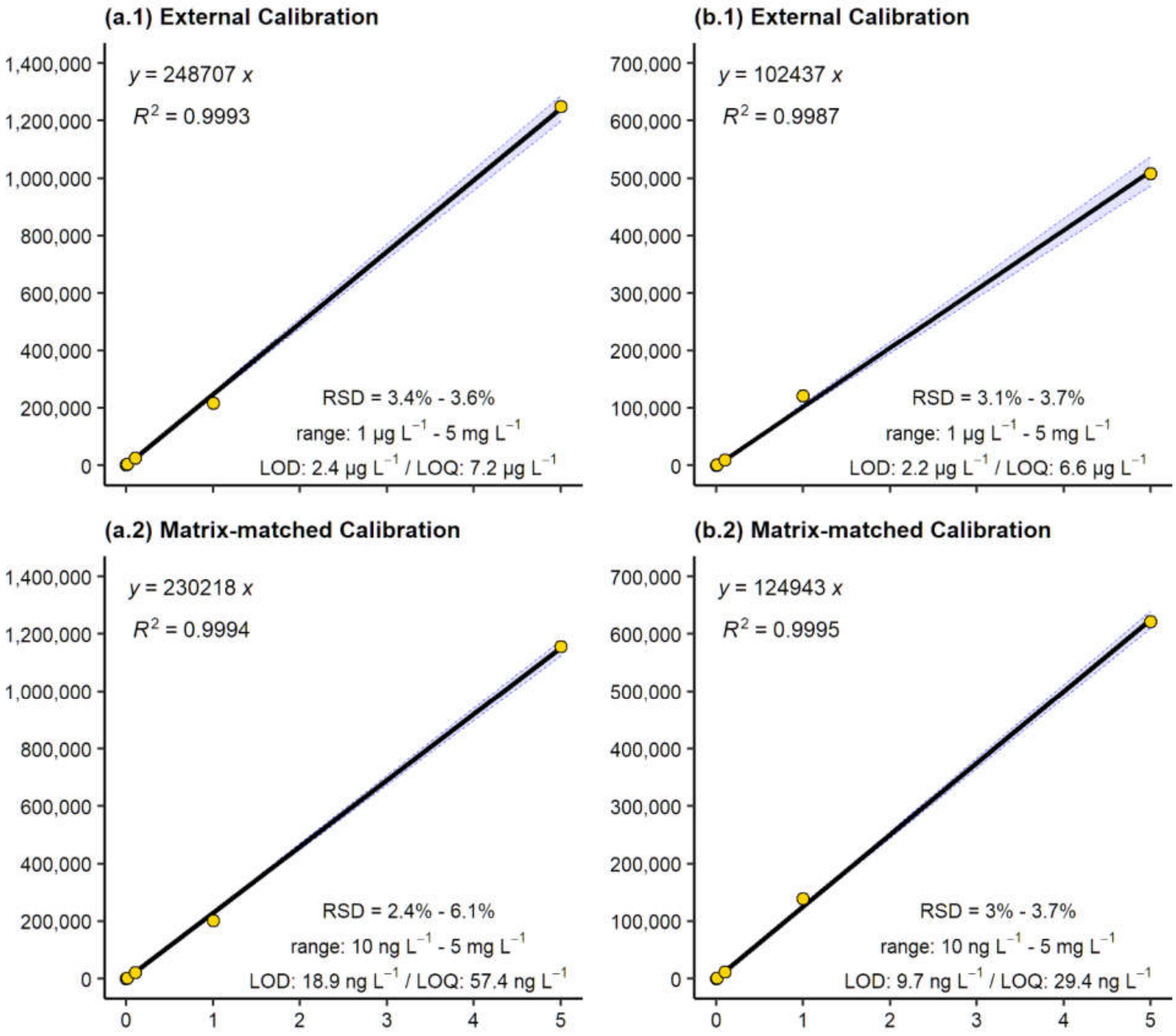

Figure S3. Calibration curves for quantification of analytes.

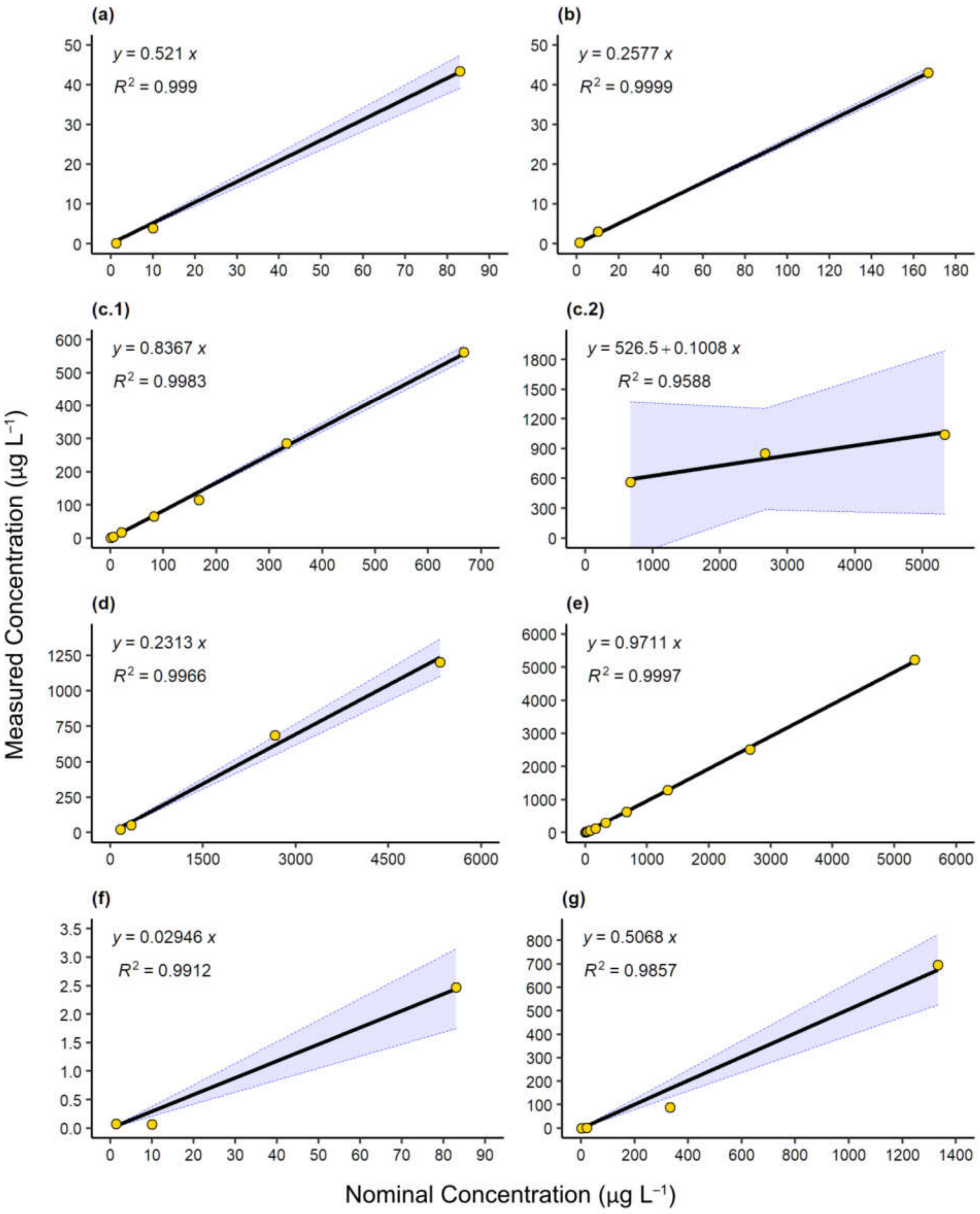

Figure S4. Linear regressions for interpolation of non-sampled exposure concentrations. Regressions including formula and coefficient of determination (R^2) shown for *L. purpurea* larval (**a**) survival and (**b**) settlement assays, *T. faulkneri* larval survival in the (**c.1**) low and (**c.2**) high concentration range and (**d**) settlement assay, (**e**) *A. millepora* larval survival, (**f**) *A. digitifera* larval survival and (**g**) recruit experiments. Shaded areas represent the 95% confidence intervals (CIs).

S2: Results

Water quality

Table S6. Water quality parameters at the start of the experiments and at the end after 48 h.

		Start Conditions		End of Experiment (48 h)	
Species	Experiment	DO (mg L^{-1})	pH	DO (mg L^{-1})	pH
L. purpurea	Survival	6.40	7.86	6.60	7.90
L. purpurea	Settlement	6.54	7.75	6.30	7.76
T. faulkneri	Survival	7.22	8.15	7.04	8.13
T. faulkneri	Settlement	7.36	8.14	7.45	8.07
A. millepora	Survival	7.23	8.12	7.21	8.26
A. digitifera	Survival	7.00	8.25	6.00	8.05

DO = dissolved oxygen.

Bioassays

Table S7. Results of larval toxicity bioassays. Results presented as median percent response and interquartile range (IQR), with nominal and measured treatment concentrations shown in μg L^{-1}.

Species	Experiment	Nominal	Measured	Endpoint	Median % Response	IQR
L. purpurea	Survival	NC	NC	mortality	0	0 – 0
		PC	PC		100	100–100
		83.3	43.5		100	100–100
		41.7	21.6		100	100–100
		20.8	10.8		0	0–0
		10.4	4		0	0–0
		5.2	2.7		0	0–10
		2.6	1.4		0	0–0
		1.3	0.1		0	0–0
A. millepora	Survival	NC	NC	mortality	0	0–0
		PC	PC		100	100–100
		5333.5	5219.4		100	100–100
		2666.8	2522.7		100	100–100
		1333.4	1289.8		20	10–60
		666.7	631.9		0	0–0
		333.3	290.3		0	0–20
		166.7	113		0	0–0
		83.3	65.5		0	0–10
		41.7	28.4		20	10–20
		20.8	14		20	10–20
		10.4	10.7		0	0–10
		5.2	4.8		0	0–0

		2.6	2.5		0	0–0
		1.3	1.2		0	0–10
A. digitifera	Survival	NC	NC	mortality	0	0–0
		PC	PC		100	100–100
		83.3	2.5		100	100–100
		41.7	1.22		100	100–100
		20.8	0.61		0	0–20
		10.4	0.067		0	0–0
		5.2	0.150		0	0–0
		2.6	0.080		0	0–0
		1.3	0.074		0	0–0
T. faulkneri	Survival	NC	NC	Mortality	0	0–0
		PC	PC		100	100–100
		5333.5	1040		80	70–90
		2666.8	684.4		60	60–70
		1333.4	661		0	0–0
		666.7	561.6		0	0–0
		333.3	285.2		0	0–0
		166.7	114.7		0	0–0
		83.3	64.7		0	0–0
		41.7	24.9		0	0–0
		20.8	17		0	0 – 0
		10.4	8.7		0	0 – 0
		5.2	4.2		0	0–10
		2.6	2.2		0	0–10
		1.3	1		0	0–0
L. purpurea	Settlement	NC	NC	Mortality	0	0–0
		PC	PC		100	0–0
		166.7	43.02		100	0–0
		83.3	21.5		0	0–30
		41.7	10.8		20	10–20
		20.8	5.4		0	0–0
		10.4	3		0	0–0
		5.2	1.3		0	0–0
		2.6	0.7		0	0–0
		1.3	0.2		0	0–0
		0	0	Settlement	80	60–90
		PC	PC		0	0–0
		166.7	43.02		0	0–0
		83.3	21.5		40	20–70
		41.7	10.8		0	0–0

		20.8	5.4		20	10–30
		10.4	3		40	30–50
		5.2	1.3		40	30–70
		2.6	0.7		80	60–90
		1.3	0.2		100	70–100
T. faulkneri	Settlement	NC	NC	Mortality	0	0–0
		PC	PC		100	100–100
		5333.5	1201.8		60	60–80
		2666.8	684.4		40	30–50
		1333.4	284.2*		0	0–10
		666.7	142.1*		0	0–0
		333.3	52.2		0	0–0
		166.7	22.3		0	0–0
		NC	NC	Settlement	100	100–100
		PC	PC		0	0–0
		5333.5	1201.8		40	20–40
		2666.8	684.4		40	20–50
		1333.4	284.2*		80	40–90
		666.7	142.1*		100	90–100
		333.3	52.2		20	10–40
		166.7	22.3		100	90–100

NC = negative control (FSW for survival and FSW and CCA chip for settlement); PC = positive control (containing 1.244 mg TBTO L^{-1} FSW, nominal).
* interpolated values based on linear regressions of nominal vs. measured concentrations (cf. Section 2.6.3).

Pairwise Comparisons

Table S8. : Detailed results of the statistical analyses for the Survival assay with *L. purpurea*.

Species (Experiment: Endpoint)	Statistical Test	Comparison [a]			Statistic	*p*-value
L. purpurea (Survival: mortality)	Shapiro – Wilk normality Test				$W = 0.427$	<0.00001
	Leven's test for homogeneity of variances				$W = 16.0$	<0.00001
	Kruskal – Wallis				$X^2(8) = 24.8$	0.0017
	Dunn's post hoc comparisons	NC	–	PC	2.556	0.011
		NC	–	83.3	2.556	0.011
		NC	–	41.7	2.556	0.011
		NC	–	20.8	0	1
		NC	–	10.4	0	1
		NC	–	5.2	0.548	0.584
		NC	–	2.6	0	1
		NC	–	1.3	0	1
		PC	–	83.3	0	1
		PC	–	41.7	0	1
		PC	–	20.8	−2.556	0.011
		PC	–	10.4	−2.556	0.011
		PC	–	5.2	−2.008	0.045
		PC	–	2.6	−2.556	0.011
		PC	–	1.3	−2.556	0.011
		83.3	–	41.7	0	1
		83.3	–	20.8	−2.556	0.011
		83.3	–	10.4	−2.556	0.011
		83.3	–	5.2	−2.008	0.045
		83.3	–	2.6	−2.556	0.011
		83.3	–	1.3	−2.556	0.011
		41.7	–	20.8	−2.556	0.011
		41.7	–	10.4	−2.556	0.011
		41.7	–	5.2	−2.008	0.045
		41.7	–	2.6	−2.556	0.011
		41.7	–	1.3	−2.556	0.011
		20.8	–	10.4	0	1
		20.8	–	5.2	0.548	0.584
		20.8	–	2.6	0	1
		20.8	–	1.3	0	1
		10.4	–	5.2	0.548	0.584
		10.4	–	2.6	0	1

		10.4	–	1.3	0	1
		5.2	–	2.6	−0.548	0.584
		5.2	–	1.3	−0.548	0.584
		2.6	–	1.3	0	1

[a] Values for multiple comparisons are expressed as nominal values

Table S9. : Detailed results of the statistical analyses for the Survival assay with *A. millepora*.

**Species (Experiment: Endpoint)**	**Statistical Test**	**Comparison** [a]			**Statistic**	***p*-value**
A. millepora (Survival: mortality)	Shapiro–Wilk normality Test				$W = 0.75$	<0.00001
	Leven's test for homogeneity of variances				$W = 9.73$	<0.00001
	Kruskal–Wallis				$X^2(14) = 31.9$	0.0041
	Dunn's post hoc comparisons	NC	–	PC	2.831	0.005
		NC	–	5333.5	2.831	0.005
		NC	–	2666.8	2.831	0.005
		NC	–	1333.4	1.538	0.124
		NC	–	666.7	0	1
		NC	–	333.3	0.751	0.452
		NC	–	166.7	0	1
		NC	–	83.3	0.594	0.552
		NC	–	41.7	1.188	0.235
		NC	–	20.8	1.188	0.235
		NC	–	10.4	0.594	0.552
		NC	–	5.2	0	1
		NC	–	2.6	0	1
		NC	–	1.3	0.594	0.552
		PC	–	5333.5	0	1
		PC	–	2666.8	0	1
		PC	–	1333.4	−1.293	0.196
		PC	–	666.7	−2.831	0.005
		PC	–	333.3	−2.080	0.038
		PC	–	166.7	−2.831	0.005
		PC	–	83.3	−2.237	0.025
		PC	–	41.7	−1.643	0.1
		PC	–	20.8	−1.643	0.1
		PC	–	10.4	−2.237	0.025
		PC	–	5.2	−2.831	0.005
		PC	–	2.6	−2.831	0.005
		PC	–	1.3	−2.237	0.025

		5333.5	–	2666.8	0	1
		5333.5	–	1333.4	−1.293	0.196
		5333.5	–	666.7	−2.831	0.005
		5333.5	–	333.3	−2.080	0.038
		5333.5	–	166.7	−2.831	0.005
		5333.5	–	83.3	−2.237	0.025
		5333.5	–	41.7	−1.643	0.1
		5333.5	–	20.8	−1.643	0.1
		5333.5	–	10.4	−2.237	0.025
		5333.5	–	5.2	−2.831	0.005
		5333.5	–	2.6	−2.831	0.005
		5333.5	–	1.3	−2.237	0.025
		2666.8	–	1333.4	−1.293	0.196
		2666.8	–	666.7	−2.831	0.005
		2666.8	–	333.3	−2.080	0.038
		2666.8	–	166.7	−2.831	0.005
		2666.8	–	83.3	−2.237	0.025
		2666.8	–	41.7	−1.643	0.1
		2666.8	–	20.8	−1.643	0.1
		2666.8	–	10.4	−2.237	0.025
		2666.8	–	5.2	−2.831	0.005
		2666.8	–	2.6	−2.831	0.005
		2666.8	–	1.3	−2.237	0.025
		1333.4	–	666.7	−1.538	0.124
		1333.4	–	333.3	−0.786	0.432
		1333.4	–	166.7	−1.538	0.124
		1333.4	–	83.3	−0.944	0.345
		1333.4	–	41.7	−0.350	0.727
		1333.4	–	20.8	−0.350	0.727
		1333.4	–	10.4	−0.944	0.345
		1333.4	–	5.2	−1.538	0.124
		1333.4	–	2.6	−1.538	0.124
		1333.4	–	1.3	−0.944	0.345
		666.7	–	333.3	0.751	0.452
		666.7	–	166.7	0	1
		666.7	–	83.3	0.594	0.552
		666.7	–	41.7	1.188	0.235
		666.7	–	20.8	1.188	0.235
		666.7	–	10.4	0.594	0.552
		666.7	–	5.2	0	1
		666.7	–	2.6	0	1

		666.7	–	1.3	0.594	0.552
		333.3	–	166.7	−0.751	0.452
		333.3	–	83.3	−0.157	0.875
		333.3	–	41.7	0.437	0.662
		333.3	–	20.8	0.437	0.662
		333.3	–	10.4	−0.157	0.875
		333.3	–	5.2	−0.751	0.452
		333.3	–	2.6	−0.751	0.452
		333.3	–	1.3	−0.157	0.875
		166.7	–	83.3	0.594	0.552
		166.7	–	41.7	1.188	0.235
		166.7	–	20.8	1.188	0.235
		166.7	–	10.4	0.594	0.552
		166.7	–	5.2	0	1
		166.7	–	2.6	0	1
		166.7	–	1.3	0.594	0.552
		83.3	–	41.7	0.594	0.552
		83.3	–	20.8	0.594	0.552
		83.3	–	10.4	0	1
		83.3	–	5.2	−0.594	0.552
		83.3	–	2.6	−0.594	0.552
		83.3	–	1.3	0	1
		41.7	–	20.8	0	1
		41.7	–	10.4	−0.594	0.552
		41.7	–	5.2	−1.188	0.235
		41.7	–	2.6	−1.188	0.235
		41.7	–	1.3	−0.594	0.552
		20.8	–	10.4	−0.594	0.552
		20.8	–	5.2	−1.188	0.235
		20.8	–	2.6	−1.188	0.235
		20.8	–	1.3	−0.594	0.552
		10.4	–	5.2	−0.594	0.552
		10.4	–	2.6	-0.594	0.552
		10.4	–	1.3	0	1
		5.2	–	2.6	0	1
		5.2	–	1.3	0.594	0.552
		2.6	–	1.3	0.594	0.552

[a] Values for multiple comparisons are expressed as nominal values.

Table S10. Detailed results of the statistical analyses for the Survival assay with *A. digitifera*.

Species (Experiment: Endpoint)	Statistical Test	Comparison [a]			Statistic	*p*-value
A. digitifera (Survival: mortality)	Shapiro – Wilk normality Test				*W* = 0.427	<0.00001
	Leven's test for homogeneity of variances				*W* = 16.0	<0.00001
	Kruskal – Wallis				$X^2(8)$ = 24.8	0.0017
	Dunn's post hoc comparisons	NC	–	PC	2.556	0.011
		NC	–	83.3	2.556	0.011
		NC	–	41.7	2.556	0.011
		NC	–	20.8	0.548	0.584
		NC	–	10.4	0	1
		NC	–	5.2	0	1
		NC	–	2.6	0	1
		NC	–	1.3	0	1
		PC	–	83.3	0	1
		PC	–	41.7	0	1
		PC	–	20.8	−2.008	0.045
		PC	–	10.4	−2.556	0.011
		PC	–	5.2	−2.556	0.011
		PC	–	2.6	−2.556	0.011
		PC	–	1.3	−2.556	0.011
		83.3	–	41.7	0	1
		83.3	–	20.8	−2.008	0.045
		83.3	–	10.4	−2.556	0.011
		83.3	–	5.2	−2.556	0.011
		83.3	–	2.6	−2.556	0.011
		83.3	–	1.3	−2.556	0.011
		41.7	–	20.8	−2.008	0.045
		41.7	–	10.4	−2.556	0.011
		41.7	–	5.2	−2.556	0.011
		41.7	–	2.6	−2.556	0.011
		41.7	–	1.3	−2.556	0.011
		20.8	–	10.4	−0.548	0.584
		20.8	–	5.2	−0.548	0.584
		20.8	–	2.6	−0.548	0.584
		20.8	–	1.3	−0.548	0.584
		10.4	–	5.2	0	1
		10.4	–	2.6	0	1
		10.4	–	1.3	0	1

		5.2	–	2.6	0	1
		5.2	–	1.3	0	1
		2.6	–	1.3	0	1

[a] Values for multiple comparisons are expressed as nominal values.

Table S11. Detailed results of the statistical analyses for the Survival assay with *T. faulkneri*.

Species (Experiment: Endpoint)	Statistical Test	Comparison [a]			Statistic	*p*-value
T. faulkneri (Survival: mortality)	Shapiro–Wilk normality Test				$W = 0.657$	<0.00001
	Leven's test for homogeneity of variances				$W = 5.57$	<0.0001
	Kruskal–Wallis				$X^2(14)=39.4$	0.0003
	Dunn's post hoc comparisons	NC	–	PC	3.218	0.001
		NC	–	5333.5	2.867	0.004
		NC	–	2666.8	2.640	0.008
		NC	–	1333.4	0	1
		NC	–	666.7	0	1
		NC	–	333.3	0	1
		NC	–	166.7	0	1
		NC	–	83.3	0	1
		NC	–	41.7	0	1
		NC	–	20.8	0	1
		NC	–	10.4	0	1
		NC	–	5.2	0.743	0.458
		NC	–	2.6	0.743	0.458
		NC	–	1.3	0	1
		PC	–	5333.5	−0.351	0.726
		PC	–	2666.8	−0.578	0.564
		PC	–	1333.4	−3.218	0.001
		PC	–	666.7	−3.218	0.001
		PC	–	333.3	−3.218	0.001
		PC	–	166.7	−3.218	0.001
		PC	–	83.3	−3.218	0.001
		PC	–	41.7	−3.218	0.001
		PC	–	20.8	−3.218	0.001
		PC	–	10.4	−3.218	0.001
		PC	–	5.2	−2.475	0.013
		PC	–	2.6	−2.475	0.013

		PC	–	1.3	−3.218	0.001
		5333.5	–	2666.8	−0.227	0.821
		5333.5	–	1333.4	−2.867	0.004
		5333.5	–	666.7	−2.867	0.004
		5333.5	–	333.3	−2.867	0.004
		5333.5	–	166.7	−2.867	0.004
		5333.5	–	83.3	−2.867	0.004
		5333.5	–	41.7	−2.867	0.004
		5333.5	–	20.8	−2.867	0.004
		5333.5	–	10.4	−2.867	0.004
		5333.5	–	5.2	−2.124	0.034
		5333.5	–	2.6	−2.124	0.034
		5333.5	–	1.3	−2.867	0.004
		2666.8	–	1333.4	−2.640	0.008
		2666.8	–	666.7	−2.640	0.008
		2666.8	–	333.3	−2.640	0.008
		2666.8	–	166.7	−2.640	0.008
		2666.8	–	83.3	−2.640	0.008
		2666.8	–	41.7	−2.640	0.008
		2666.8	–	20.8	−2.640	0.008
		2666.8	–	10.4	−2.640	0.008
		2666.8	–	5.2	−1.898	0.058
		2666.8	–	2.6	−1.898	0.058
		2666.8	–	1.3	−2.640	0.008
		1333.4	–	666.7	0	1
		1333.4	–	333.3	0	1
		1333.4	–	166.7	0	1
		1333.4	–	83.3	0	1
		1333.4	–	41.7	0	1
		1333.4	–	20.8	0	1
		1333.4	–	10.4	0	1
		1333.4	–	5.2	0.743	0.458
		1333.4	–	2.6	0.743	0.458
		1333.4	–	1.3	0	1
		666.7	–	333.3	0	1
		666.7	–	166.7	0	1
		666.7	–	83.3	0	1
		666.7	–	41.7	0	1
		666.7	–	20.8	0	1
		666.7	–	10.4	0	1
		666.7	–	5.2	0.743	0.458

		666.7	–	2.6	0.743	0.458
		666.7	–	1.3	0	1
		333.3	–	166.7	0	1
		333.3	–	83.3	0	1
		333.3	–	41.7	0	1
		333.3	–	20.8	0	1
		333.3	–	10.4	0	1
		333.3	–	5.2	0.743	0.458
		333.3	–	2.6	0.743	0.458
		333.3	–	1.3	0	1
		166.7	–	83.3	0	1
		166.7	–	41.7	0	1
		166.7	–	20.8	0	1
		166.7	–	10.4	0	1
		166.7	–	5.2	0.743	0.458
		166.7	–	2.6	0.743	0.458
		166.7	–	1.3	0	1
		83.3	–	41.7	0	1
		83.3	–	20.8	0	1
		83.3	–	10.4	0	1
		83.3	–	5.2	0.743	0.458
		83.3	–	2.6	0.743	0.458
		83.3	–	1.3	0	1
		41.7	–	20.8	0	1
		41.7	–	10.4	0	1
		41.7	–	5.2	0.743	0.458
		41.7	–	2.6	0.743	0.458
		41.7	–	1.3	0	1
		20.8	–	10.4	0	1
		20.8	–	5.2	0.743	0.458
		20.8	–	2.6	0.743	0.458
		20.8	–	1.3	0	1
		10.4	–	5.2	0.743	0.458
		10.4	–	2.6	0.743	0.458
		10.4	–	1.3	0	1
		5.2	–	2.6	0	1
		5.2	–	1.3	−0.743	0.458
		2.6	–	1.3	−0.743	0.458

[a] Values for multiple comparisons are expressed as nominal values.

Table S12. Detailed results of the statistical analysis for the endpoint 'mortality' in the Settlement assay with *L. purpurea.*

Species (Experiment: Endpoint)	Statistical Test	Comparison [a]			Statistic	*p*-value
L. purpurea (Settlement: mortality)	Shapiro–Wilk normality Test				$W = 0.542$	<0.00001
	Leven's test for homogeneity of variances				$W = 14.93$	<0.00001
	Kruskal–Wallis				$X^2(9) = 25.0$	0.003
	Dunn's post hoc comparisons	NC	–	PC	2.849	0.004
		NC	–	166.7	2.849	0.004
		NC	–	83.3	0.748	0.454
		NC	–	41.7	1.324	0.186
		NC	–	20.8	0	1
		NC	–	10.4	0	1
		NC	–	5.2	0	1
		NC	–	2.6	0	1
		NC	–	1.3	0	1
		PC	–	166.7	0	1
		PC	–	83.3	−2.100	0.036
		PC	–	41.7	−1.525	0.127
		PC	–	20.8	−2.849	0.004
		PC	–	10.4	−2.849	0.004
		PC	–	5.2	−2.849	0.004
		PC	–	2.6	−2.849	0.004
		PC	–	1.3	−2.849	0.004
		166.7	–	83.3	−2.100	0.036
		166.7	–	41.7	−1.525	0.127
		166.7	–	20.8	−2.849	0.004
		166.7	–	10.4	−2.849	0.004
		166.7	–	5.2	−2.849	0.004
		166.7	–	2.6	−2.849	0.004
		166.7	–	1.3	−2.849	0.004
		83.3	–	41.7	0.575	0.565
		83.3	–	20.8	−0.748	0.454
		83.3	–	10.4	−0.748	0.454
		83.3	–	5.2	−0.748	0.454
		83.3	–	2.6	−0.748	0.454
		83.3	–	1.3	−0.748	0.454
		41.7	–	20.8	−1.324	0.186
		41.7	–	10.4	−1.324	0.186
		41.7	–	5.2	−1.324	0.186

		41.7	–	2.6	−1.324	0.186
		41.7	–	1.3	−1.324	0.186
		20.8	–	10.4	0	1
		20.8	–	5.2	0	1
		20.8	–	2.6	0	1
		20.8	–	1.3	0	1
		10.4	–	5.2	0	1
		10.4	–	2.6	0	1
		10.4	–	1.3	0	1
		5.2	–	2.6	0	1
		5.2	–	1.3	0	1
		2.6	–	1.3	0	1

[a] Values for multiple comparisons are expressed as nominal values.

Table S13. Detailed results of the statistical analysis for the endpoint 'settlement' in the Settlement assay with *L. purpurea.*

**Species (Experiment: Endpoint)**	**Statistical Test**	**Comparison** [a]			**Statistic**	***p*-value**
L. purpurea (Settlement: settlement)	Shapiro–Wilk normality Test				$W = 0.947$	0.1432
	Leven's test for homogeneity of variances				$W = 3.1$	0.017
	Kruskal–Wallis				$X^2(9) = 20.6$	0.015
	Dunn's post hoc comparisons	NC	–	PC	−2.453	0.014
		NC	–	166.7	−2.453	0.014
		NC	–	83.3	−0.842	0.400
		NC	–	41.7	−2.453	0.014
		NC	–	20.8	−1.539	0.124
		NC	–	10.4	−0.770	0.442
		NC	–	5.2	−0.505	0.614
		NC	–	2.6	0	1
		NC	–	1.3	0.192	0.847
		PC	–	166.7	0	1
		PC	–	83.3	1.611	0.107
		PC	–	41.7	0	1
		PC	–	20.8	0.914	0.361
		PC	–	10.4	1.683	0.092
		PC	–	5.2	1.948	0.051
		PC	–	2.6	2.453	0.014
		PC	–	1.3	2.645	0.008
		166.7	–	83.3	1.611	0.107

		166.7	–	41.7	0	1
		166.7	–	20.8	0.914	0.361
		166.7	–	10.4	1.683	0.092
		166.7	–	5.2	1.948	0.051
		166.7	–	2.6	2.453	0.014
		166.7	–	1.3	2.645	0.008
		83.3	–	41.7	−1.611	0.107
		83.3	–	20.8	−0.697	0.486
		83.3	–	10.4	0.072	0.942
		83.3	–	5.2	0.337	0.736
		83.3	–	2.6	0.842	0.400
		83.3	–	1.3	1.034	0.301
		41.7	–	20.8	0.914	0.361
		41.7	–	10.4	1.683	0.092
		41.7	–	5.2	1.948	0.051
		41.7	–	2.6	2.453	0.014
		41.7	–	1.3	2.645	0.008
		20.8	–	10.4	0.770	0.442
		20.8	–	5.2	1.034	0.301
		20.8	–	2.6	1.539	0.124
		20.8	–	1.3	1.731	0.083
		10.4	–	5.2	0.265	0.791
		10.4	–	2.6	0.770	0.442
		10.4	–	1.3	0.962	0.336
		5.2	–	2.6	0.505	0.614
		5.2	–	1.3	0.697	0.486
		2.6	–	1.3	0.192	0.847

[a] Values for multiple comparisons are expressed as nominal values.

Table S14. Detailed results of the statistical analysis for the endpoint 'mortality' in the Settlement assay with *T. faulkneri.*

Species (Experiment: Endpoint)	Statistical Test	Comparison [a]			Statistic	*p*-value
T. faulkneri (Settlement: Mortality)	Shapiro–Wilk normality Test				$W = 0.778$	<0.001
	Leven's test for homogeneity of variances				$W = 6.16$	0.001
	Kruskal–Wallis				$X^2(7) = 21.6$	0.003
	Dunn's post hoc comparisons	NC	–	PC	2.913	0.004
		NC	–	5333.5	2.460	0.014
		NC	–	2666.8	1.877	0.061
		NC	–	1333.4	0.518	0.605
		NC	–	666.7	0	1
		NC	–	333.3	0	1
		NC	–	166.7	0	1
		PC	–	5333.5	−0.453	0.650
		PC	–	2666.8	−1.036	0.300
		PC	–	1333.4	−2.395	0.017
		PC	–	666.7	−2.913	0.004
		PC	–	333.3	−2.913	0.004
		PC	–	166.7	−2.913	0.004
		5333.5	–	2666.8	−0.583	0.560
		5333.5	–	1333.4	−1.942	0.052
		5333.5	–	666.7	−2.460	0.014
		5333.5	–	333.3	−2.460	0.014
		5333.5	–	166.7	−2.460	0.014
		2666.8	–	1333.4	−1.359	0.174
		2666.8	–	666.7	−1.877	0.061
		2666.8	–	333.3	−1.877	0.061
		2666.8	–	166.7	−1.877	0.061
		1333.4	–	666.7	−0.518	0.605
		1333.4	–	333.3	−0.518	0.605
		1333.4	–	166.7	−0.518	0.605
		666.7	–	333.3	0	1
		666.7	–	166.7	0	1
		333.3	–	166.7	0	1

[a] Values for multiple comparisons are expressed as nominal values.

Table S15. Detailed results of the statistical analysis for the endpoint 'settlement' in the Settlement assay with *T. faulkneri*.

**Species (Experiment: Endpoint)**	**Statistical Test**	**Comparison** [a]			**Statistic**	***p*-value**
T. faulkneri (Settlement: Settlement)	Shapiro–Wilk normality Test				$W = 0.778$	<0.001
	Leven's test for homogeneity of variances				$W = 6.16$	0.001
	Kruskal–Wallis				$X^2(7) = 21.6$	0.003
	Dunn's post hoc comparisons	NC	–	PC	−2.955	0.003
		NC	–	5333.5	−2.239	0.025
		NC	–	2666.8	−2.090	0.037
		NC	–	1333.4	−1.313	0.189
		NC	–	666.7	−0.328	0.743
		NC	–	333.3	−2.209	0.027
		NC	–	166.7	−0.328	0.743
		PC	–	5333.5	0.716	0.474
		PC	–	2666.8	0.866	0.387
		PC	–	1333.4	1.642	0.101
		PC	–	666.7	2.627	0.009
		PC	–	333.3	0.746	0.456
		PC	–	166.7	2.627	0.009
		5333.5	–	2666.8	0.149	0.881
		5333.5	–	1333.4	0.925	0.355
		5333.5	–	666.7	1.910	0.056
		5333.5	–	333.3	0.030	0.976
		5333.5	–	166.7	1.910	0.056
		2666.8	–	1333.4	0.776	0.438
		2666.8	–	666.7	1.761	0.078
		2666.8	–	333.3	−0.119	0.905
		2666.8	–	166.7	1.761	0.078
		1333.4	–	666.7	0.985	0.325
		1333.4	–	333.3	−0.896	0.371
		1333.4	–	166.7	0.985	0.325
		666.7	–	333.3	−1.881	0.060
		666.7	–	166.7	0	1
		333.3	–	166.7	1.881	0.060

[a] Values for multiple comparisons are expressed as nominal values.

Biological Endpoints

Table S16. Biological endpoints after 48 h of BP3 exposure. Comparison of measured vs. nominal LC/EC_{50} values in µg L^{-1} derived from probit regression analysis including lower and upper 95% confidence intervals in brackets shown for each species and experiment.

	Survival Assay		Settlement Assay			
	LC_{50}		LC_{50}		EC_{50}	
**Species**	**Measured**	**Nominal**	**Measured**	**Nominal**	**Measured**	**Nominal**
L. purpurea	13.47 [10.58, 17.14]	26.56 [21.27, 33.17]	23.35 [18.85, 28.93]	90.41 [72.92, 112.10]	1.84 [0.97, 3.47]	7.65 [4.23, 13.85]
T. faulkneri	2951.24 [813.63, 10705]	5623.06 [1978, 15987]	799.84 [603.80, 1060]	3390.05 [2564, 4483]	298.92 [122.40, 730.00]	1400.08 [675.61, 2901]
A. millepora	1042.31 [543.82, 1998]	1158.27 [616.34, 2177]	-	-	-	-
A. digitifera	0.75 [0.66, 0.87]	25.82 [22.51, 29.61]	-	-	-	-

Table S17. Statistical comparison of LC_{50s} between species in the Survival experiment performed by ratio test.

Comparison			LC_{50} (µg L^{-1})		Ratio Test statistic		
**Species 1**	**Species 2**	**Measured/ Nominal**	**Species 1**	**Species 2**	**SE**	**Z-statistic**	***p*-value**
L. purpurea	*T. faulkneri*	Measured	13.5	2951	0.095	24.7	3.1×10^{-134}
		Nominal	26.6	5623	0.071	32.9	6.9×10^{-237}
L. purpurea	*A. digitifera*	Measured	13.5	0.75	0.255	4.91	9.1×10^{-7}
		Nominal	26.6	25.8	0.041	0.30	0.764
L. purpurea	*A. millepora*	Measured	13.5	1042	0.067	28.1	1.6×10^{-173}
		Nominal	26.6	1158	0.057	28.6	2.2×10^{-180}
A. millepora	*A. digitifera*	Measured	1042	0.75	0.255	12.3	7.9×10^{-35}
		Nominal	1158	25.8	0.050	32.7	3.3×10^{-235}
A. millepora	*T. faulkneri*	Measured	1042	2951	0.095	4.75	2.0×10^{-6}
		Nominal	1158	5623	0.077	8.94	4.0×10^{-19}
T. faulkneri	*A. digitifera*	Measured	2951	0.75	0.264	13.6	3.0×10^{-42}
		Nominal	5623	25.8	0.065	35.8	4.7×10^{-280}

Table S18. Statistical comparison of LC_{50}s between species in the Settlement experiment performed by ratio test.

Comparison			LC_{50} (μg L^{-1})		Ratio Test statistic		
**Species 1**	**Species 2**	**Measured/ Nominal**	**Species 1**	**Species 2**	**SE**	**Z-statistic**	***p*-value**
L. purpurea	*T. faulkneri*	Measured	23.4	800	0.041	37.6	0
		Nominal	90.4	3390	0.030	52.4	0

Table S19. Statistical comparison of EC_{50}s between species in the Settlement experiment performed by ratio test.

Comparison			EC_{50} (μg L^{-1})		Ratio Test statistic		
**Species 1**	**Species 2**	**Measured/ Nominal**	**Species 1**	**Species 2**	**SE**	**Z-statistic**	***p*-value**
L. purpurea	*T. faulkneri*	Measured	1.84	299	0.583	4.1	3.9×10^{-5}
		Nominal	7.65	1400	0.157	14.4	6.2×10^{-47}

Table S20. Statistical comparison of LC_{50} vs. EC_{50} values between species in the Settlement experiment performed by ratio test.

Comparison			LC/EC (μg L^{-1})		Ratio Test statistic		
**Species 1**	**Species 2**	**Measured/ Nominal**	**LC_{50}**	**EC_{50}**	**SE**	**Z-statistic**	***p*-value**
L. purpurea	*L. purpurea*	Measured	23.4	1.84	0.533	2.07	0.0385
		Nominal	90.4	7.65	0.151	7.12	1.1×10^{-12}
T. faulkneri	*T. faulkneri*	Measured	800	299	0.083	5.17	2.4×10^{-7}
		Nominal	3390	1400	0.054	7.08	1.4×10^{-12}

Table S21. Statistical comparison of LC_{50s} between the same species in the Survival vs. the Settlement experiment performed by ratio test.

Comparison			LC_{50} (µg L^{-1})		Ratio Test statistic		
**Survival assay**	**Settlement assay**	**Measured/ Nominal**	**Species 1**	**Species 2**	**SE**	**Z-statistic**	***p*-value**
L. purpurea	*L. purpurea*	Measured	13.5	23.4	0.059	4.07	4.6×10^{-5}
		Nominal	26.6	90.4	0.042	12.6	2.8×10^{-36}
T. faulkneri	*T. faulkneri*	Measured	2951	800	0.085	6.67	2.6×10^{-11}
		Nominal	5263	3390	0.064	3.42	6.2×10^{-4}

Table S22. Statistical comparison of the most sensitive biological endpoints in the Survival vs. the Settlement experiment performed by ratio test.

Comparison			LC/EC (µg L^{-1})		Ratio Test statistic		
**Species 1**	**Species 2**	**Measured/ Nominal**	$LC_{50\ [survival]}$	$EC_{50\ [settlement]}$	**SE**	**Z-statistic**	***p*-value**
A. digitifera	*L. purpurea*	Measured	0.75	1.84	0.588	0.659	0.510
		Nominal	25.8	7.65	0.150	3.52	4.4×10^{-4}

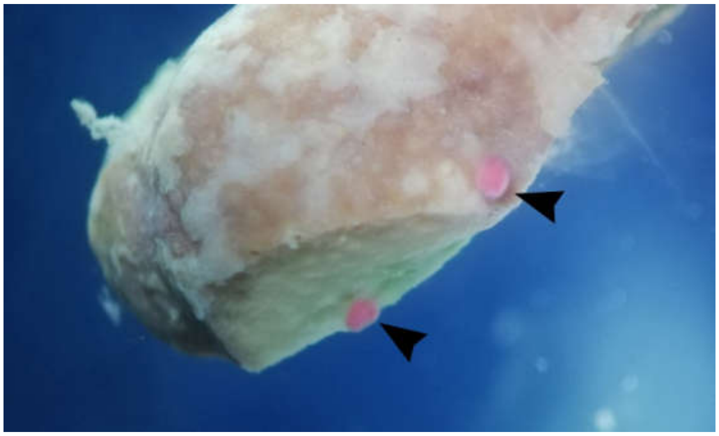

Figure S5. CCA induced stress response in planula larvae of *L. purpurea*. Planula turned pink (arrows) and died within 48 h, likely indicating potential microbial defense reactions towards the coral larvae.

S3. Larvae Bioassays with Additional Chemical Compounds

S3.1 Material and Methods

Table S23. Chemical compounds tested in additional ecotoxicological experiments and their relevant physiochemical properties.

Compound	Formula	CAS No	Structure	Water solubility	Log K_{ow}	Density (g cm^{-3})	Comments	Acquired From
Benzophenone-3; 98% [BP3]	$C_{14}H_{12}O_3$	131-57-7	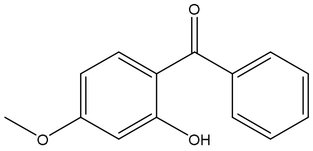	[a]6 mg L^{-1} (25 °C)	[a]3.45 (40 °C)	[a]1.43 (20 °C)	UVA, UVB	Sigma Aldrich Chemistry
Octocrylene [OCR]	$C_{24}H_{27}NO_2$	6197-30-	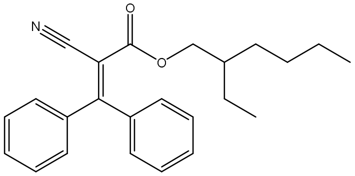	[a]40 µg L^{-1}	[a]6.1 (23 °C)	[a]1.05 (25 °C)	UVB	BASF
Ethyl-hexyl-methoxy-cinnamate [EHMC]	$C_{18}H_{26}O_3$	5466-77-3	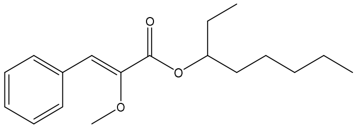	[a]41 µg L^{-1}	[a]6.0 (23 °C)	[a]1.01 (20 °C)	UVB	BASF
Cinnamic acid benzyl ester; 98% [CABE]	$C_{16}H_{14}O_2$	103-41-3	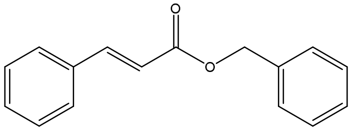	[a]3.6 mg L^{-1} (20 °C)	[a]4.18 (23.7 °C)	[a]1.12		abcr GmbH

Quercetin; ≥95% [QUE]	$C_{15}H_{10}O_7$	117-39-5	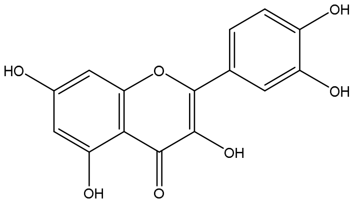	[b]Practically insoluble	[b]1.99 (25 °C)	[c]1.36	UVA	Sigma Aldrich Chemistry
Coumarin; ≥99% [COUM]	$C_9H_6O_2$	91-64-5	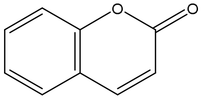	[a]1900 mg L^{-1} (20 °C)	[a]1.39	[a]0.935 (20 °C)	UVB	Sigma Aldrich Chemistry
trans-Anethole; 99% [ANT]	$C_{10}H_{12}O$	4180-23-8	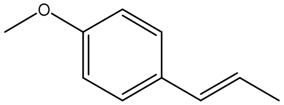	[a]111 mg L^{-1} (25 °C)	[a]3.39	[a]0.987 (20 °C)		Sigma Aldrich Chemistry
L-Phenylalanine; ≥98% [PHE]	$C_9H_{11}NO_2$	63-91-2	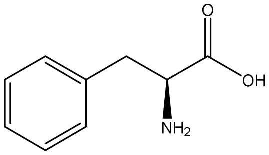	[a]25 g L^{-1}	[a]–1.52 (20 °C)	[a]1.34 (20 °C)		Sigma Aldrich Chemistry
Bis(tri-n-butyltin) oxide; 97% [TBTO]	$C_{24}H_{54}OSn_2$	56-35-9	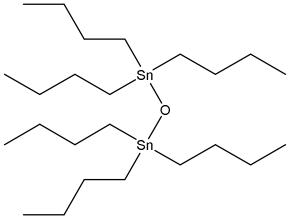	[a]71.2 mg L^{-1} (20 °C)	[a]2.2	[a]1.17 (28 °C)		abcr GmbH

Diuron; ≥98% [DCMU]	$C_9H_{10}Cl_2N_2O$	330-54-1	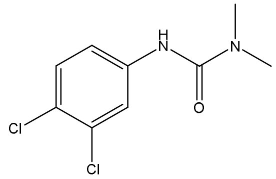	[a]29 mg L^{-1} (20 °C)	[a]2.84 (20 °C)	[a]1.454 (20 °C)	Sigma Aldrich Chemistry
Copper(I) chloride; ≥99% [CuCl]	ClCu	7758-89-6	Cu^{+} Cl^{-}	[h]47 mg L^{-1} (20 °C)	[d]-0.26	[a]4.14	Sigma Aldrich Chemistry

Sources: [a][1]; [b](obtained from Sigma Aldrich safety data sheets); [c][2]; [d][3].

Table S24. Nominal concentrations of saturated stock solutions used for serial dilutions in ecotoxicological experiments. Concentrations are not adjusted to water solubility limits.

Compound.	Stock solution ($mg\ L^{-1}$ FSW)
Benzophenone-3 (BP3)	12
Octocrylene (OCR)	84
Ethyl-hexyl-methoxy-cinnamate (EHMC)	99.82
Quercetin (QUE)	100
Coumarin (COUM)	100
Bis(tri-n-butyltin) oxide	140
Diuron (DCMU)	72
Copper(I) chloride (CuCl)	94
Phenylalanine (PHE)	100
Anethole (ANT)	98.8
Cinnamic acid benzyl ester (CABE)	100

S3.1 Results

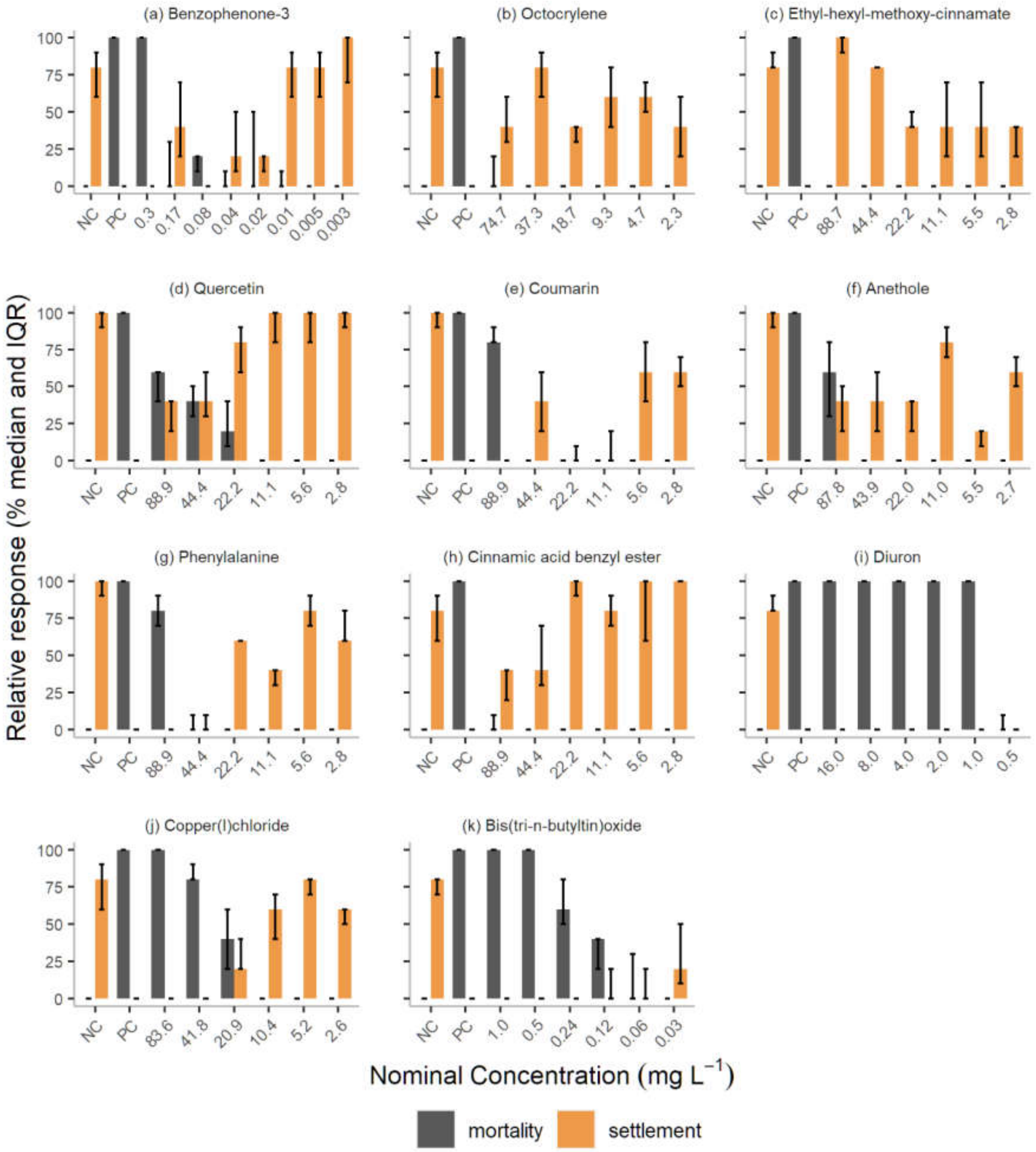

Figure S6. Results of additional larval stage settlement assays with *L. purpurea.* Bars indicate relative median mortality and settlement rates and interquartile range (IQR) after 48 h (n = 3 replicates, 5 planulae each). Note: Concentrations are not adjusted to water solubility limits.

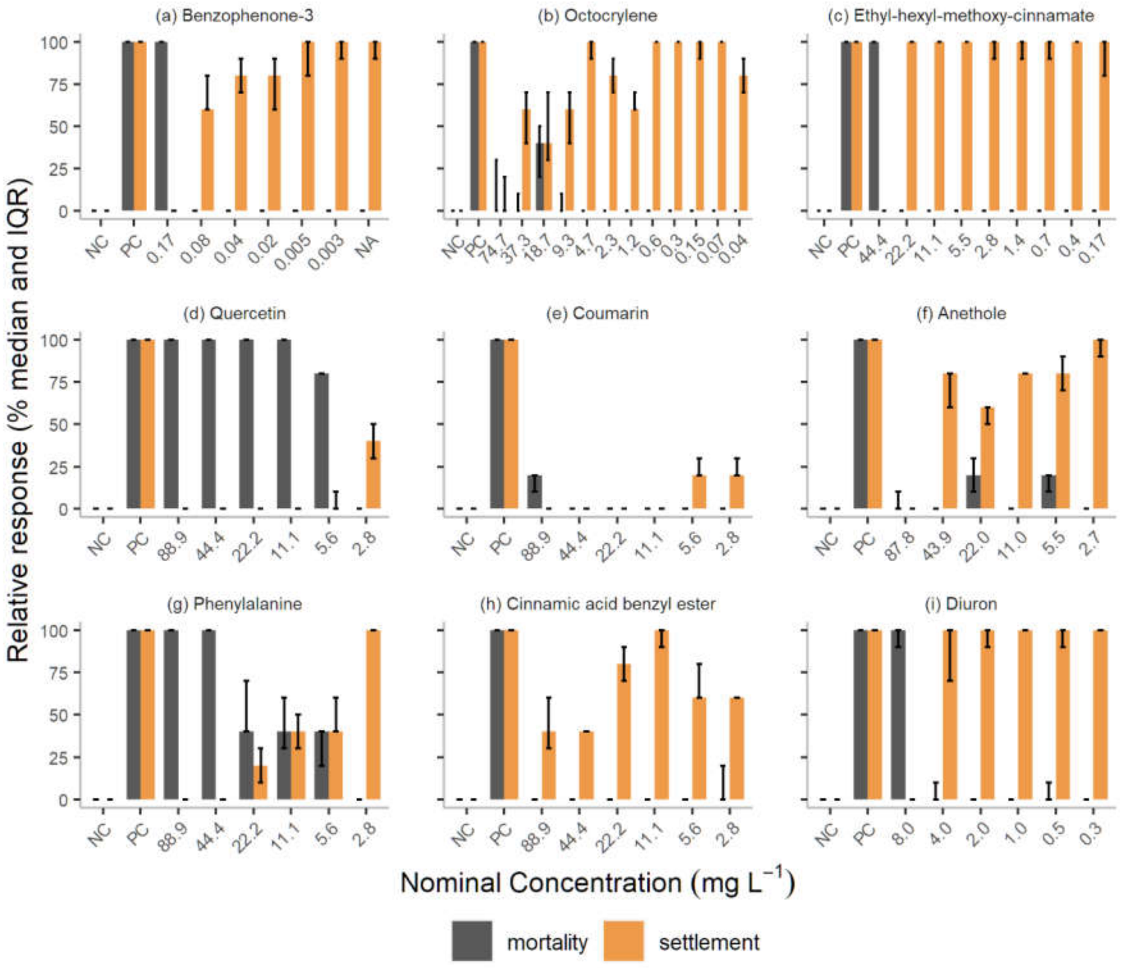

Figure S7. Results of additional larval stage settlement assays with *A. digitifera*. Bars indicate relative median mortality and settlement rates and interquartile range (IQR) after 48 h (n = 3 replicates, 5 planulae each). Concentrations are not adjusted to water solubility limits.

S4. Bioassay with Early Post-Settlement Recruits of *A. digitifera*

S4.1 Material and Methods

Fecund colonies (n = 8, ca 25 cm in diameter) of the hermatypic broadcast spawning coral *A. digitifera* were collected from the reef crest at Pago Bay (13°25'35"N, 144°47'47"E) prior to the spawning event and held in 4000 L tanks sustained by an open natural seawater flow-through system (cf. Table S25 for details). During the spawning in July, gamete bundles were collected from the water surface, and subsequently, sperm and eggs were mechanically separated. Cross-fertilization was achieved by mixing gametes from several colonies. After fertilization, embryos were transferred into larval culture tanks from where planula larvae were collected for the larval assays. The remaining larvae were put into tanks containing chips made from coral rubble overgrown with crustose coralline algae (CCA) as settlement substrate. Upon settlement, the recruits were reared for two months before being used in the recruit bioassays. A CCA chip containing three individual primary polyps was placed in each well (Figure S8) (6-well cell culture plate; Polystyrene; Product No. 92406; TPP Techno Plastic Products AG, Switzerland) assess polyp reactivity in the recruit assay was assessed semiquantitatively by gently tipping both; a 200 µL pipette tip was used to gently induce a response on tentacles and the mouth of the primary polyp.

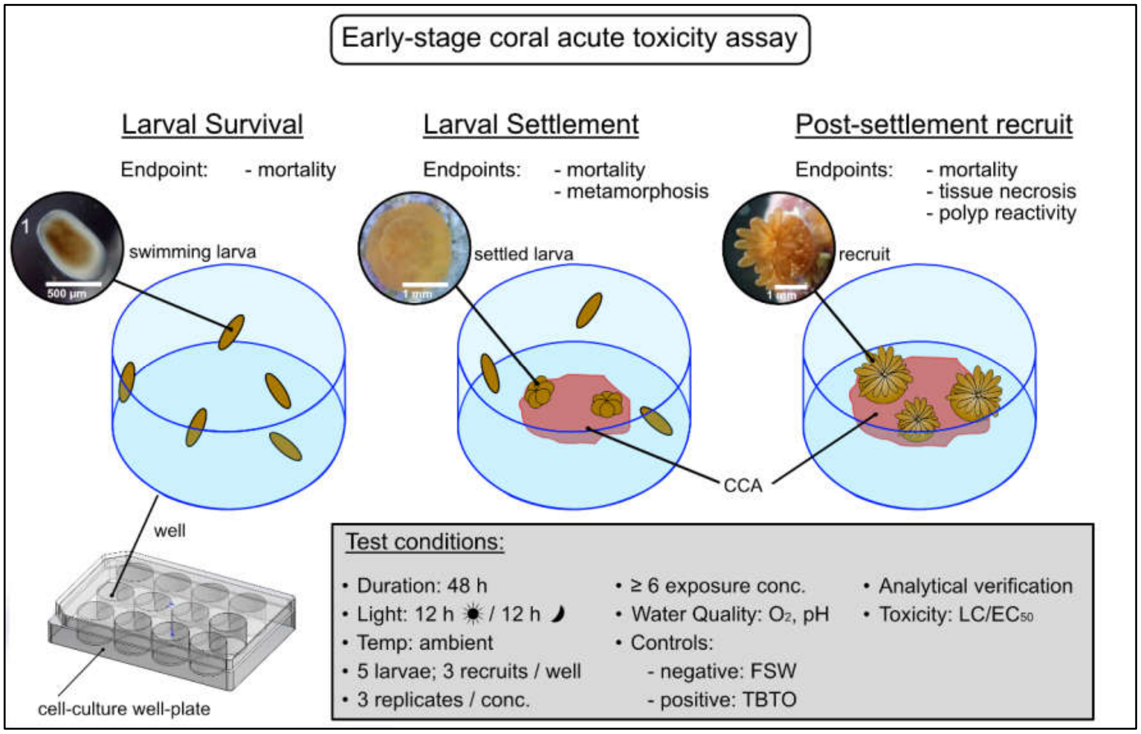

Figure S8. Experimental setup of the bioassays including recruits.

Table S25. Overview of experimental conditions for recruit bioassay.

**Experiment**	**Post-Settlement Recruit Survival**
**Species**	*A. digitifera*
**Well plate**	6-well cell culture plate; Polystyrene (Product No. 92406; TPP Techno Plastic Products AG, Switzerland)
**Water**	
- Origin (cf. Table S1 for details)	Natural seawater
- Pre-treatment for stock solutions and bioassays	0.22 µm filter sterilized
Abbreviation	FSW
**Test Media:**	
- Target chemical	BP3
- Negative (media) control	FSW + CCA[1]
- Positive control	1.2 mg TBTO L^{-1} FSW
- Volume/well	9 mL
**Settlement inducer**	CCA[1] (2 months before test)
**Replicates**	3
**Pseudo-replicates**	3 polyps on CCA (~1 cm^3) chip / well
**Exposure concentrations (nominal)**	11 (2-fold serial dilution ranging from 1333.4–1.3 µg/L) + controls
**Incubation conditions:**	
- Duration	48 h
- Light source	Artificial (white light source)
- Light cycle: day/night	12h/12h
- Light intensity	4300 LUX<br>[80 µmol m^{-2} s^{-1}]
- Temperature	29–30
**Toxicological endpoints:**	- Tissue necrosis<br>- Polyp reactivity<br>- Mortality

[1]CCA = crustose coralline algae (*Hydrolithon reinboldii*) originated from Luminao Reef, Guam (13°27'56''N, 144°38'48''E).

S4.2 Results

For the assessment of the suitability of the proposed test system for a later stage of the life history of coral, 2-month old *A. digitifera* recruits were exposed to BP3, also for 48 h. In this assay, tissue necrosis and polyp reactivity were investigated as endpoints in addition to mortality. DO levels were 6 mg L^{-1} at test start and after 48 h; pH was 8.15 before and 8.11 at termination of the assay. Polyps in the negative control were in a healthy condition at the end of the assay with full reactivity and no signs of tissue necrosis, while all acroporids died in the positive control with complete tissue necrosis, therefore

satisfying the validity criteria. In general, there was a decreasing pattern of mortality associated with tissue necrosis with decreasing exposure concentrations, while polyp reactivity increased (Figure S9). The LOEC was at 666.7 µg L^{-1} (measured 337.7 µg L^{-1}) for all three endpoints (Dunn's Test: p values ≤ 0.014, Table 26). All acroporids in this treatment showed tissue necrosis, 67% (IQR 50–83%) died, and33% (IQR 17–33%) were still reactive. Polyp reactivity in the highest concentration (1333.4 µg L^{-1} nominal; 695.9 µg L^{-1} measured) was identical to the latter, while mortality was slightly higher at around 67% (IQR 66–83%), and all recruits (median, IQR 83–100%) showed signs of tissue necrosis. Some minor effects (with no statistical relevance; Dunn's Test: p-values > 0.05, Table S27) were observed below the LOEC down to 20.8 µg L^{-1} (measured 2.6 µg L^{-1}), and none in the lower treatments.

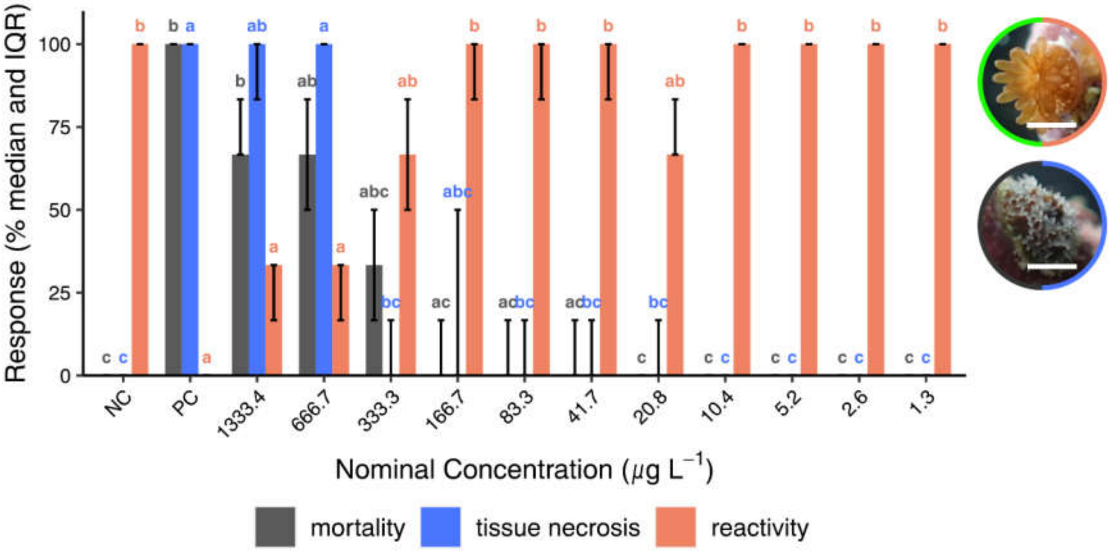

Figure S9. Results of the bioassay with *Acropora digitifera* recruits after 48 h BP3 exposure. Relative median mortality, tissue necrosis and polyp reactivity and interquartile range (IQR) are shown for each treatment. Letters indicate significance groups obtained from post hoc Dunn's test, with same letters indicating no significant differences. Green/pink circle: healthy recruit with reactive tentacles; grey/blue circle: recruit showing severe tissue necrosis and mortality; Scale bar = 1 mm.

Recruits of *A. digitifera* were less susceptible compared to the larval stage of the same species. In fact, the recruit LC_{50} was 218.64 µg L^{-1} and thus, over 300-fold higher than the larval LC_{50}. The most affected endpoint was tissue necrosis with an EC_{50} of 132.32 µg L^{-1}, while polyp reactivity was less affected in a similar range than mortality (Figure S10).

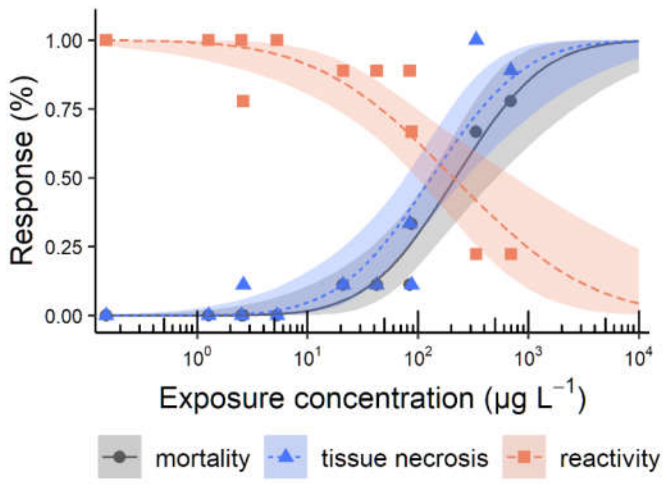

Figure S10. Concentration (measured) – Response relationship of the recruit experiment for *A. digitifera* recruits after 48 h BP3 exposure. Probit regressions with shaded areas indicating 95% confidence intervals illustrated for three endpoints.

Table S26. Results of toxicity bioassay with *A. digitifera* recruits. Presented as median percent response and interquartile range (IQR), with nominal and measured treatment concentrations shown in µg L^{-1}.

Nominal	Measured	Endpoint	Median % Response	IQR
NC	NC	mortality	0	0–0
PC	PC		100	100–100
1333.4	695.9		67	66–83
666.7	337.9*		67	50–83
333.3	87.8		33	17–50
166.7	84.5*		0	0–17
83.3	42.2*		0	0–17
41.7	21.1*		0	0–17
20.8	2.6		0	0–0
10.4	5.3*		0	0–0
5.2	2.6*		0	0–0
2.6	1.3*		0	0–0
1.3	0.15		0	0–0
NC	NC	Tissue necrosis	0	0–0
PC	PC		100	100–100
1333.4	695.9		100	83–100
666.7	337.9*		100	100–100
333.3	87.8		0	0–17
166.7	84.5*		0	0–50
83.3	42.2*		0	0–17
41.7	21.1*		0	0–17
20.8	2.6		0	0–17
10.4	5.3*		0	0–0
5.2	2.6*		0	0–0
2.6	1.3*		0	0–0
1.3	0.15		0	0–0
NC	NC	Reactivity	100	100–100

PC	PC		0	0–0
1333.4	695.9		33	17–33
666.7	337.9*		33	17–33
333.3	87.8		67	50–84
166.7	84.5*		100	83–100
83.3	42.2*		100	83–100
41.7	21.1*		100	83–100
20.8	2.6		67	67–83
10.4	5.3*		100	100–100
5.2	2.6*		100	100–100
2.6	1.3*		100	100–100
1.3	0.15		100	100–100

NC = negative control; PC = positive control containing 0.7 mg TBTO L^{-1} FSW, nominal * interpolated values based on linear regressions of nominal vs. measured concentrations (cf. Section 2.6.3).

Table S27. Multiple comparisons for the recruit bioassay.

**Species (Experiment: Endpoint)**	**Statistical Test**	**Comparison** [a]			**Statistic**	***p*-value**
A, digitifera (Recruit: Mortality)	Shapiro – Wilk normality Test				$W = 0.81$	<0.0001
	Leven’s test for homogeneity of variances				$W = 3.73$	0.002
	Kruskal – Wallis				$X^2(12) = 30.5$	0.002
	Dunn’s post hoc comparisons	NC	−	1.3	0	1.000
		NC	−	10.4	0	1.000
		NC	−	1333.4	2.637	0.008
		NC	−	166.7	0.628	0.530
		NC	−	2.6	0	1.000
		NC	−	20.8	0	1.000
		NC	−	333.3	1.444	0.149
		NC	−	41.7	0.628	0.530
		NC	−	5.2	0.000	1.000
		NC	−	666.7	2.449	0.014
		NC	−	83.3	0.628	0.530
		NC	−	PC	3.014	0.003
		1.3	−	10.4	0	1.000
		1.3	−	1333.4	2.637	0.008
		1.3	−	166.7	0.628	0.530
		1.3	−	2.6	0	1.000
		1.3	−	20.8	0	1.000
		1.3	−	333.3	1.444	0.149
		1.3	−	41.7	0.628	0.530
		1.3	−	5.2	0.000	1.000
		1.3	−	666.7	2.449	0.014
		1.3	−	83.3	0.628	0.530

		1.3	−	PC	3.014	0.003
		10.4	−	1333.4	2.637	0.008
		10.4	−	166.7	0.628	0.530
		10.4	−	2.6	0	1.000
		10.4	−	20.8	0	1.000
		10.4	−	333.3	1.444	0.149
		10.4	−	41.7	0.628	0.530
		10.4	−	5.2	0	1.000
		10.4	−	666.7	2.449	0.014
		10.4	−	83.3	0.628	0.530
		10.4	−	PC	3.014	0.003
		1333.4	−	166.7	−2.009	0.045
		1333.4	−	2.6	−2.637	0.008
		1333.4	−	20.8	−2.637	0.008
		1333.4	−	333.3	−1.193	0.233
		1333.4	−	41.7	−2.009	0.045
		1333.4	−	5.2	−2.637	0.008
		1333.4	−	666.7	−0.188	0.851
		1333.4	−	83.3	−2.009	0.045
		1333.4	−	PC	0.377	0.706
		166.7	−	2.6	−0.628	0.530
		166.7	−	20.8	−0.628	0.530
		166.7	−	333.3	0.816	0.414
		166.7	−	41.7	0	1.000
		166.7	−	5.2	−0.628	0.530
		166.7	−	666.7	1.821	0.069
		166.7	−	83.3	0	1.000
		166.7	−	PC	2.386	0.017
		2.6	−	20.8	0	1.000
		2.6	−	333.3	1.444	0.149
		2.6	−	41.7	0.628	0.530
		2.6	−	5.2	0	1.000
		2.6	−	666.7	2.449	0.014
		2.6	−	83.3	0.628	0.530
		2.6	−	PC	3.014	0.003
		20.8	−	333.3	1.444	0.149
		20.8	−	41.7	0.628	0.530
		20.8	−	5.2	0	1.000
		20.8	−	666.7	2.449	0.014
		20.8	−	83.3	0.628	0.530
		20.8	−	PC	3.014	0.003
		333.3	−	41.7	−0.816	0.414
		333.3	−	5.2	−1.444	0.149
		333.3	−	666.7	1.005	0.315
		333.3	−	83.3	−0.816	0.414
		333.3	−	PC	1.570	0.117
		41.7	−	5.2	−0.628	0.530

		41.7	–	666.7	1.821	0.069
		41.7	–	83.3	0	1.000
		41.7	–	PC	2.386	0.017
		5.2	–	666.7	2.449	0.014
		5.2	–	83.3	0.628	0.530
		5.2	–	PC	3.014	0.003
		666.7	–	83.3	−1.821	0.069
		666.7	–	PC	0.565	0.572
		83.3	–	PC	2.386	0.017
A, digitifera (Recruit: Tissue necrosis)	Shapiro–Wilk normality Test				$W = 0.792$	<0.0001
	Leven's test for homogeneity of variances				$W = 11.2$	<0.0001
	Kruskal – Wallis				$X^2(12)=28.4$	0.005
	Dunn's post hoc comparisons	NC	–	1.3	0	1.000
		NC	–	10.4	0	1.000
		NC	–	1333.4	2.567	0.010
		NC	–	166.7	0.926	0.354
		NC	–	2.6	0	1.000
		NC	–	20.8	0.610	0.542
		NC	–	333.3	0.610	0.542
		NC	–	41.7	0.610	0.542
		NC	–	5.2	0	1.000
		NC	–	666.7	2.778	0.005
		NC	–	83.3	0.610	0.542
		NC	–	PC	2.778	0.005
		1.3	–	10.4	0	1.000
		1.3	–	1333.4	2.567	0.010
		1.3	–	166.7	0.926	0.354
		1.3	–	2.6	0	1.000
		1.3	–	20.8	0.610	0.542
		1.3	–	333.3	0.610	0.542
		1.3	–	41.7	0.610	0.542
		1.3	–	5.2	0	1.000
		1.3	–	666.7	2.778	0.005
		1.3	–	83.3	0.610	0.542
		1.3	–	PC	2.778	0.005
		10.4	–	1333.4	2.567	0.010
		10.4	–	166.7	0.926	0.354
		10.4	–	2.6	0	1.000
		10.4	–	20.8	0.610	0.542
		10.4	–	333.3	0.610	0.542
		10.4	–	41.7	0.610	0.542
		10.4	–	5.2	0	1.000
		10.4	–	666.7	2.778	0.005
		10.4	–	83.3	0.610	0.542
		10.4	–	PC	2.778	0.005
		1333.4	–	166.7	-1.642	0.101
		1333.4	–	2.6	-2.567	0.010

		1333.4	–	20.8	-1.957	0.050
		1333.4	–	333.3	−1.957	0.050
		1333.4	–	41.7	−1.957	0.050
		1333.4	–	5.2	−2.567	0.010
		1333.4	–	666.7	0.210	0.833
		1333.4	–	83.3	−1.957	0.050
		1333.4	–	PC	0.210	0.833
		166.7	–	2.6	−0.926	0.354
		166.7	–	20.8	−0.316	0.752
		166.7	–	333.3	−0.316	0.752
		166.7	–	41.7	−0.316	0.752
		166.7	–	5.2	−0.926	0.354
		166.7	–	666.7	1.852	0.064
		166.7	–	83.3	−0.316	0.752
		166.7	–	PC	1.852	0.064
		2.6	–	20.8	0.610	0.542
		2.6	–	333.3	0.610	0.542
		2.6	–	41.7	0.610	0.542
		2.6	–	5.2	0	1.000
		2.6	–	666.7	2.778	0.005
		2.6	–	83.3	0.610	0.542
		2.6	–	PC	2.778	0.005
		20.8	–	333.3	0	1.000
		20.8	–	41.7	0	1.000
		20.8	–	5.2	−0.610	0.542
		20.8	–	666.7	2.168	0.030
		20.8	–	83.3	0	1.000
		20.8	–	PC	2.168	0.030
		333.3	–	41.7	0	1.000
		333.3	–	5.2	−0.610	0.542
		333.3	–	666.7	2.168	0.030
		333.3	–	83.3	0	1.000
		333.3	–	PC	2.168	0.030
		41.7	–	5.2	−0.610	0.542
		41.7	–	666.7	2.168	0.030
		41.7	–	83.3	0	1.000
		41.7	–	PC	2.168	0.030
		5.2	–	666.7	2.778	0.005
		5.2	–	83.3	0.610	0.542
		5.2	–	PC	2.778	0.005
		666.7	–	83.3	−2.168	0.030
		666.7	–	PC	0	1.000
		83.3	–	PC	2.168	0.030
A, digitifera (Recruit: Reactivity)	Shapiro–Wilk normality Test				$W = 0.878$	0.0006
	Leven's test for homogeneity of variances				$W = 4.53$	0.0006
	Kruskal–Wallis				$X^2(12) = 12.0$	0.002

		NC	−	1.3	0	1.000
		NC	−	10.4	0	1.000
		0	−	1333.4	−2.623	0.009
		0	−	166.7	−0.585	0.559
		0	−	2.6	0	1.000
		NC	−	20.8	−1.170	0.242
		NC	−	333.3	−1.392	0.164
		NC	−	41.7	−0.585	0.559
		NC	−	5.2	0	1.000
		NC	−	666.7	−2.623	0.009
		NC	−	83.3	−0.585	0.559
		NC	−	PC	−3.026	0.002
		1.3	−	10.4	0	1.000
		1.3	−	1333.4	−2.623	0.009
		1.3	−	166.7	−0.585	0.559
		1.3	−	2.6	0	1.000
		1.3	−	20.8	−1.170	0.242
		1.3	−	333.3	−1.392	0.164
		1.3	−	41.7	−0.585	0.559
		1.3	−	5.2	0	1.000
		1.3	−	666.7	−2.623	0.009
		1.3	−	83.3	−0.585	0.559
		1.3	−	PC	−3.026	0.002
	Dunn's post hoc comparisons	10.4	−	1333.4	−2.623	0.009
		10.4	−	166.7	−0.585	0.559
		10.4	−	2.6	0	1.000
		10.4	−	20.8	−1.170	0.242
		10.4	−	333.3	−1.392	0.164
		10.4	−	41.7	−0.585	0.559
		10.4	−	5.2	0	1.000
		10.4	−	666.7	−2.623	0.009
		10.4	−	83.3	−0.585	0.559
		10.4	−	PC	−3.026	0.002
		1333.4	−	166.7	2.038	0.042
		1333.4	−	2.6	2.623	0.009
		1333.4	−	20.8	1.452	0.146
		1333.4	−	333.3	1.231	0.218
		1333.4	−	41.7	2.038	0.042
		1333.4	−	5.2	2.623	0.009
		1333.4	−	666.7	0	1.000
		1333.4	−	83.3	2.038	0.042
		1333.4	−	PC	−0.403	0.687
		166.7	−	2.6	0.585	0.559
		166.7	−	20.8	−0.585	0.559
		166.7	−	333.3	−0.807	0.420
		166.7	−	41.7	0.000	1.000
		166.7	−	5.2	0.585	0.559

		166.7	–	666.7	−2.038	0.042
		166.7	–	83.3	0	1.000
		166.7	–	PC	−2.441	0.015
		2.6	–	20.8	−1.170	0.242
		2.6	–	333.3	−1.392	0.164
		2.6	–	41.7	−0.585	0.559
		2.6	–	5.2	0	1.000
		2.6	–	666.7	−2.623	0.009
		2.6	–	83.3	−0.585	0.559
		2.6	–	PC	−3.026	0.002
		20.8	–	333.3	−0.222	0.824
		20.8	–	41.7	0.585	0.559
		20.8	–	5.2	1.170	0.242
		20.8	–	666.7	−1.452	0.146
		20.8	–	83.3	0.585	0.559
		20.8	–	PC	−1.856	0.063
		333.3	–	41.7	0.807	0.420
		333.3	–	5.2	1.392	0.164
		333.3	–	666.7	−1.231	0.218
		333.3	–	83.3	0.807	0.420
		333.3	–	PC	−1.634	0.102
		41.7	–	5.2	0.585	0.559
		41.7	–	666.7	−2.038	0.042
		41.7	–	83.3	0	1.000
		41.7	–	PC	−2.441	0.015
		5.2	–	666.7	−2.623	0.009
		5.2	–	83.3	−0.585	0.559
		5.2	–	PC	−3.026	0.002
		666.7	–	83.3	2.038	0.042
		666.7	–	PC	−0.403	0.687
		83.3	–	PC	−2.441	0.015

[a] Values for multiple comparisons are expressed as nominal values.

References

1. ECHA The REACH registration dossier Available online: https://echa.europa.eu/information-on-chemicals/registered-substances (accessed on 6 May 2022).
2. ChemicalBook Chemical Book Available online: https://www.chemicalbook.com/productindex_en.aspx (accessed on 25 May 2020).
3. ChemSpider ChemSpider. Search and share chemistry Available online: http://www.chemspider.com/ (accessed on 24 May 2020).
4. Zhang, Y.; Lee, H.K. Determination of ultraviolet filters in water samples by vortex-assisted dispersive liquid–liquid microextraction followed by gas chromatography–mass spectrometry. *J. Chromatogr. A* **2012**, *1249*, 25–31, doi:10.1016/j.chroma.2012.06.019.
